# A CADASIL *NOTCH3* mutation leads to clonal hematopoiesis and expansion of *Dnmt3a-R878H* hematopoietic clones

**DOI:** 10.1038/s41375-024-02464-8

**Published:** 2024-11-13

**Authors:** Raúl Sánchez-Lanzas, Justin Barclay, Alexandros Hardas, Foteini Kalampalika, Amanda Jiménez-Pompa, Paolo Gallipoli, Miguel Ganuza

**Affiliations:** 1 Centre for Hemato-Oncology, Barts Cancer Institute, Queen Mary University of London, London, UK; 2 Royal Veterinary College, Hertfordshire, UK; 3 Francis Crick Institute, London, UK

**Keywords:** Haematopoietic stem cells, Haematopoiesis, Haematological cancer

## Abstract

Clonal hematopoiesis (CH) is nearly universal in the elderly. The molecular and cellular mechanisms driving CH and the clinical consequences of carrying clonally derived mutant mature blood cells are poorly understood. We recently identified a C223Y mutation in the extracellular domain (ECD) of NOTCH3 as a putative CH driver in mice. Provocatively, germline *NOTCH3* ECD mutations perturbing cysteine numbers cause Cerebral Autosomal Dominant Arteriopathy with Subcortical Infarcts and Leukoencephalopathy (CADASIL), a type of vascular dementia, suggesting an unexpected link between CADASIL and CH. Here, we formally demonstrated that mouse hematopoietic stem and progenitor cells (HSPCs) expressing CADASIL-related *NOTCH3*^*C455R*^ exhibit a proliferative advantage resulting in robust cellular expansion in vivo and in vitro. Co-expression of *NOTCH3*^*C455R*^ and *Dnmt3a*^*R878H*^, homologous to a frequent human CH mutation, increased the fitness of *NOTCH3*^*C455R*^ HSPCs, demonstrating their functional cooperation. Surprisingly, the presence of *NOTCH3*^*C455R*^ hematopoietic cells supported the expansion of *Dnmt3a*^*R878H*^ HSPCs in a non-cell autonomous fashion in vivo, strongly suggesting that CADASIL patients and asymptomatic carriers can be highly predisposed to *DNMT3A*^*R882H*^-driven CH. Considering that CADASIL-related *NOTCH3* mutations are more frequent in the general population than anticipated (~1 carrier in 400 people), the effect of these *NOTCH3* mutations on CH development should be considered.

## Introduction

Human life expectancy has significantly increased over the last century, especially in developed countries, due to advances in modern medicine and technology. Therefore, the incidence of age-related diseases, including cancers and dementia, are growing in our societies [1]. Ageing in the hematopoietic system is accompanied by a loss of adaptative immunity, higher incidence of anemia and myeloid malignancies [2]. This arises from a functional decrease in the hematopoietic stem cell (HSC) activity and the acquisition of mutations by HSCs and progenitor cells (HSPCs) [2]. During leukemogenesis, HSPCs sequentially acquire mutations that lead to leukemia. Early detection of pre-leukemic clones and the development of preventive treatments to block their progression into leukemia are attracting growing attention. Broad implementation of next generation DNA sequencing has exposed the presence of clones contributing to a large proportion (≥2%) of the peripheral blood (PB) in asymptomatic individuals [3]. This phenomenon, known as clonal hematopoiesis (CH), was first documented in the PB of female patients due to skewed patterns of lyonization in the X chromosome [3]. Large clones are considered CHIP (CH of indeterminate potential) when harboring leukemic driver mutations [3]. Commonly mutated genes in CHIP include *DNMT3A*, *TET2*, *ASXL1*, *PPM1D* and *JAK2*. CHIP patients are reportedly at a ~10-fold and ~3-fold higher risk of developing leukemia and cardiovascular conditions, respectively [4–7], although recent reports have questioned the correlation with cardiovascular disease [7]. CH is widely observed in geriatric patients (>70% of people older than 90 years display CH). This condition is not exclusive to aged individuals and confers similar risks to young carriers [3–6].

Despite recent extensive efforts, the plethora of cellular and molecular mechanisms driving CH emergence, selection and evolution over an individual’s life are poorly understood. CH may result from neutral drift or mutational/epimutational events conferring a selective advantage [8]. Furthermore, the functional and clinical consequences of harboring most of these CH clones are unexplored. Only recently have mouse studies revealed that *Tet2*^*−/−*^ or *Jak2*^*V617F*^ myeloid cells promote cardiovascular damage by sustaining local inflammation [9, 10]. However, the consequences of carrying other CH clones (including those not yet classified as CHIP) are unknown.

To better understand the mechanisms that confer a selective advantage during CH, we recently performed an in vivo functional screen [11] where genetically labeled bone marrow (BM) was serially transplanted. This extreme stress led to the clonal collapse of transplanted BM, inducing CH. Here, we detected 27 mutations using single nucleotide variants [11]. Six of these mutations occurred in regions conserved in the human genome and associated with malignancy: *Bcl11b, Hist1h2ac, Npy2r, Notch3, Ptprr* and *Top2b*. Particularly, *NOTCH3* activating mutations constitute oncogenic drivers of various human cancers, including T-cell acute lymphoblastic leukemia (T-ALL) [12–14]. Activating NOTCH mutations bypass the need for NOTCH-ligand binding to activate NOTCH signaling [13, 15]. NOTCH pathway is central in coordinating cell-to-cell interactions, dictates cell fate during development and is commonly altered in cancer development [16, 17]. Interestingly, *NOTCH3* mutations were recently identified in clonally expanded human esophageal tissue [18]. Furthermore, germline mutations in *NOTCH3* affecting the number of cysteines in its extracellular domain (ECD) cause Cerebral Autosomal Dominant Arteriopathy with Subcortical Infarcts and Leukoencephalopathy (CADASIL). CADASIL is the most frequent monogenic condition leading to small-vessel disease. CADASIL patients develop general arteriopathy which affects severely the brain vessels [19]. This causes migraines, ischemic stroke, vascular dementia and finally, acute encephalopathy and cognitive impairment [20]. Currently, there are no therapeutic treatments for CADASIL [21]. Provocatively, our CH mouse screen found a *Notch3*^*C223Y*^ mutation in a conserved Ca^+2^ binding domain of the *Notch3* locus (Cys222 in the human protein), suggesting a putative link between CH and CADASIL [11]. Importantly, this *NOTCH3*^*C222Y*^ mutation has been described in four CADASIL patients [22]. The effect of CADASIL-related *NOTCH3* mutations on HSPC fitness and CH is completely unknown. Thus, this study aimed to determine the role of CADASIL-*NOTCH3* mutations in CH.

NOTCH receptors are single-pass transmembrane proteins containing 30–36 epidermal growth factor-like repeat domains (EGFR). Membrane-tethered NOTCH-ligands (e.g. JAGGED) in juxtaposing cells bind to NOTCH receptors in interacting cells inducing a conformational change. This exposes NOTCH motifs that become accessible for protease-mediated cleavage by gamma-secretase and ADAM proteases releasing NOTCH intracellular domain that relocates into the nucleus activating NOTCH transcriptional pathway [16]. CADASIL-related *NOTCH3* mutations induce protein misfolding and aggregation, favored by enhanced interactions with extracellular matrix components in the brain including vitronectin and TIMP3 [19, 21, 23]. The expression of CADASIL-related NOTCH3 mutant forms in vascular smooth muscle cells (VSMCs) results in blood vessel degeneration [24]. Yet, the biological defects responsible for CADASIL are poorly understood and the impact of CADASIL-*NOTCH3* mutations in other cellular components, as hematopoietic cells, is largely unexplored.

To tackle this, we combined a CRE recombinase (CRE)-inducible *R26-NOTCH3*^*floxC455R-GFP*^ (shorthanded as *NOTCH3*^*C455R*^) knock-in mouse [20] with the HSPC-specific *HSC-Scl-CRE-ERT* allele [25] to allow inducible expression of human NOTCH3-C455R and GFP in HSPCs. This mutation, which also affects the number of cysteines in NOTCH3-ECD, is particularly interesting as patients carrying a *NOTCH3*^*C455R*^ germline mutation experience severe CADASIL with early stroke onset (median age = 31 years) and widespread white-matter defects [26]. Via this model, we demonstrated that *NOTCH3*^*C455R*^ expression confers a selective advantage to HSPCs in vitro and in vivo.

*DNMT3A* somatic mutations in the hematopoietic system are broadly observed in the general population (~35% of CH cases) [3, 27]. Specifically, *DNMT3A*^*R882H*^ constitutes the most common CH mutation in humans [3, 27]. Importantly, recent studies have shown that although the frequency of CADASIL clinical cases is ~4 in 100,000, the actual prevalence of *NOTCH3* variants affecting the number of cysteines is much higher than expected in the general population (~1 in 400) and associated with increased incidence of vascular dementia. [19, 28–31] The phenotype observed in carriers of CADASIL-*NOTCH3* variants ranges from the development of CADASIL to non-penetrance [31].

Thus, considering the high frequency of *DNMT3A* somatic mutations and of CADASIL germline mutations in the general population, we reasoned that they very likely co-occur and/or co-exist. Hence, we investigated the combinatorial effect of CADASIL-*NOTCH3* and *DNMT3A* mutations in the fitness of HSPCs. Intriguingly, co-expression of *NOTCH3*^*C455R*^ and *Dnmt3a*^*R878H*^, homologous to human *DNMT3A*^*R882H*^, resulted in faster accumulation of double mutant hematopoietic cells in vitro, indicating a molecular cooperation between these mutations. Additionally, *NOTCH3-C455R* expressing hematopoietic cells conferred a selective advantage to *Dnmt3a*^*R878H*^ mutant HSPCs in a non-cell autonomous fashion, suggesting that CADASIL patients (at least *NOTCH3*^*C455R*^ carriers) would be more prone to the expansion of *DNMT3A*^*R882H*^ clones than the general population. Thus, our data indicate that NOTCH3-C455R can induce CH via two mechanisms: a cell autonomous effect driving a proliferative advantage and a non-cell autonomous process triggering the expansion of *DNMT3A*^*R882H*^ mutant cells.

## Material and methods

### Mice

All experiments involving mice were performed under Queen Mary University of London Veterinary oversight with UK Home Office authorization and complied with all relevant ethical regulations regarding animal research. Details on employed strains are under Supplementary Material and Methods.

### Tamoxifen delivery in vivo

To activate CRE in vivo, 8–10-week-old mice were treated with TAM by oral gavage as indicated in Supplementary Materials and Methods and within each figure.

### Genotyping

PCRs for genotyping the different mouse strains were performed employing GoTaq-G2-Flexi DNA Polymerase (Promega). PCR conditions and primers are detailed in Supplementary Materials and Methods and Supplementary Table 1.

### PB analysis

Mouse PB was collected and stained as referred in Supplementary Materials and Methods.

### Bone marrow analysis

BM was harvested from femurs, tibias, pelvic bones and spines of mice by crushing. Cells were stained and analyzed to identify HSPCs populations as described in Supplementary Materials and Methods.

### Cell cycle analysis

After staining for surface antigens, cells were fixed and permeabilized with Cytofix/Cytoperm-Fixation/Permeabilization kit (BD-Biosciences, San Diego, CA) and stained with Ki67-e660 (SolA15, Invitrogen) and 0.02 µg/µL DAPI. Details in Supplementary Materials and Methods.

### Apoptosis analysis

Following staining for surface proteins with fluorescently labeled antibodies, cells were stained with AnnexinV-APC (BioLegend) and 0.1 µg/mL DAPI.

### Isolation of hematopoietic stem and progenitor cells (HSPCs)

c-Kit^+^ BM-derived cells were enriched magnetically and then stained with fluorescently conjugated antibodies and sorted. Details in Supplementary Material and Methods.

### Statistics and reproducibility

Quantitative data are reported as means ± standard deviation. Statistical significance was determined using a two-tailored Student’s *t*-test and Tukey’s multiple comparison test at a level of 0.05. *p* values < 0.05 were considered statistically significant. Details in Supplementary Material and Methods.

### HSPC cell cultures

Mouse HSPCS were cultured in serum-free expansion medium as previously described [32]. Further details in Supplementary Material and Methods.

### Quantitative real-time PCR (qRT-PCR) analysis

qRT-PCR was performed employing SYBR Green as previously described [33]. Details and primers are specified in Supplementary Materials and Methods and Supplementary Table 2.

### Transcriptional profiling (RNAseq) and mRNA expression analysis

mRNA libraries were sequenced on an Illumina NovaSeq6000 platform. mRNA expression and gene set enrichment analyses were performed using NovoMagic platform. Details in Supplementary Materials and Methods.

### Colony genotyping of Dnmt3a^R878H^ mutant colonies

Individual colonies expanded in vitro from sorted HSPCs were genotyped as previously described [34] to detect *Dnmt3a-WT*, *Dnmt3a-R878H* and floxed (fl-*Dnmt3a-R878H)* alleles. Details in Supplementary Material and Methods and Supplementary Table 3.

## Results

### NOTCH3-C455R expression confers a proliferative advantage to HSPCs in vitro

Our prior functional screen revealed *Notch3*^*C223Y*^ as a putative CH driver mutation [11]. The effect of CADASIL-related *NOTCH3* mutations on HSPC fitness and CH is completely unknown. To determine its relevance, we first evaluated *Notch3* pattern of expression in the mouse hematopoietic hierarchy by qRT-PCR on sorted mouse HSCs, multipotent progenitors (MPPs), hematopoietic progenitor cells-1 (HPC-1); and HPC-2 [35], and in myeloid (CD11b^+^Gr1^−^ and CD11b^−^Gr1^+^) and lymphoid (B- and T-) blood cells (Supplementary Fig. 1A). qRT-PCR showed that *Notch3* mRNA is significantly enriched in HSCs and MPPs compared to differentiated blood cells (Supplementary Fig. 1A) suggesting a role for NOTCH3 in HSC/MPP biology. Taking advantage of publicly available human databases, we observed that *NOTCH3* mRNA is upregulated in HSPCs (CD133^+^CD34^dim^ and CD38^−^CD34^+^) relative to most other blood compartments across the human hematopoietic system (Supplementary Fig. 1B) further supporting that NOTCH3 plays a function in HSC biology.

**Fig. 1:**
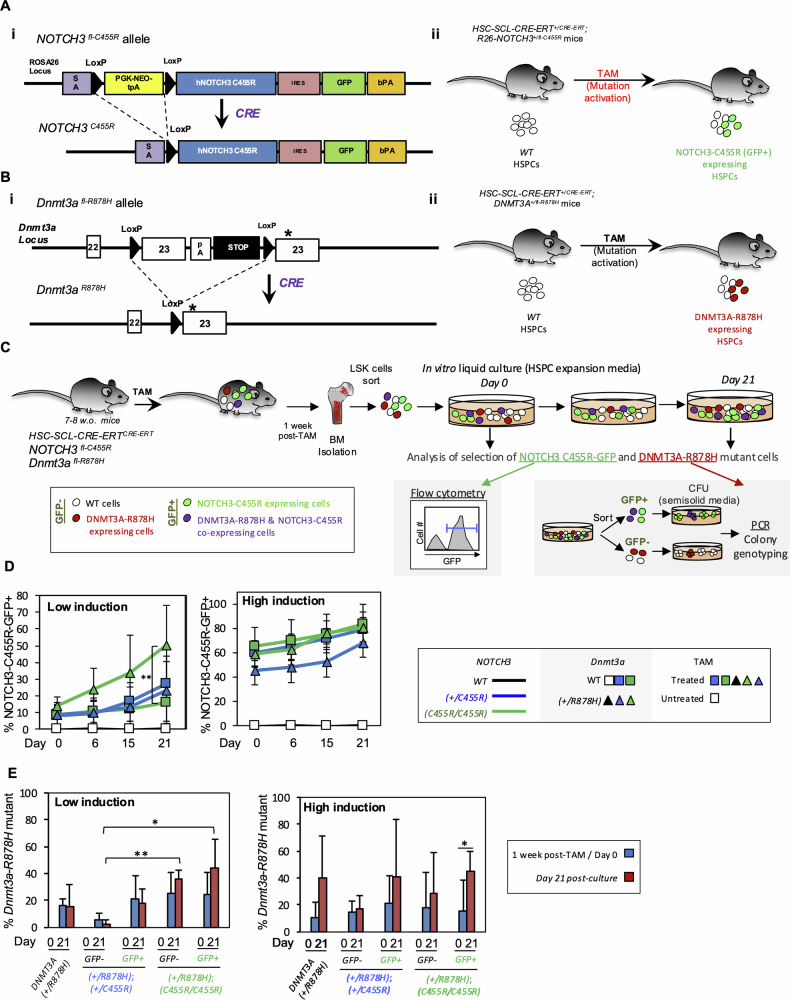
NOTCH3-C455R expression confers a fitness advantage to HSPCs in vitro. **A** R26-*NOTCH3*^*fl-C455R*^ conditional mouse model. **Ai** Schematic of the *NOTCH3*^*fl-C455R*^ allele. A transcriptional STOP cassette blocks NOTCH3-C455R and GFP expression in the initial unrecombined conformation. **Aii** CRE-inducible *R26-NOTCH3* ^*fl-C455R*^ allele was combined with the *HSC-Scl-CRE-ERT* allele to allow tamoxifen (TAM)-inducible expression of NOTCH3-C455R and GFP in HSPCs upon TAM treatment in mice. **B** *Dnmt3a*^*fl-R878H*^ conditional mouse model. **Bi** Schematic of the *Dnmt3a*^*fl-R878H*^ allele**. Bii** TAM treatment of *HSC-Scl-CRE-ERT*^*+/CRE*^*; Dnmt3a*^*+/R878H*^ mice leads to DNMT3A-R878H expression in HSPCs. **C** Schematic on experimental approach. HSPCs isolated from the bone marrow of TAM-treated mice harboring *HSC-Scl-CRE-ERT and NOTCH3*^*fl-C455R*^ in a *Dnmt3a* wt and *Dnmt3a*^*fl-R878H*^ background, were cultured in vitro in expansion media to study cell competition. NOTCH3-C455R expressing cells can be tracked by simultaneous GFP expression by flow cytometry. To evaluate the accumulation of *Dnmt3a*^*R878H*^ HSPCs, GFP^+^ and GFP^−^ HSPCs were sorted and plated in semi-solid media before (day 0) and after cell culture (21 days) to determine clonal expansion by genotyping of colonies for *Dnmt3a*^*R878H*^. **D** Two TAM treatments were administered in vivo to induce low and high % of *NOTCH3*^*C455R*^ and *Dnmt3a*^*R878H*^ HSPCs. In vitro cell culture of HSPCs derived from TAM-treated *HSC-Scl-CRE-ERT*^*+/CRE*^*;R26-NOTCH3*^*+/C455R*^ and *HSC-Scl-CRE-ERT*^*+/CRE*^*;R26-NOTCH3*^*C455R/C455R*^ mice resulted in the progressive accumulation of NOTCH3-C455R expressing cells (GFP^+^ cells), demonstrating that NOTCH3-C455R expression increases the fitness of HSPCs. At low induction, the presence of *Dnmt3a*^*R878H*^ enhanced the growth of GFP^+^ *NOTCH3*^*C455R/C455R*^ cells showing cooperation among both mutations. **E** Genotyping of colonies pre- and post-culture from (**D**) revealed that by 21 days post-culture *Dnmt3a*^*R878H*^ provides a selective advantage to double mutant GFP^+^ *NOTCH3*^*C455R/C455R*^ HSPCs, suggesting a functional cooperation among both mutations. Data at day 0 in (**E**) is the same as at 1-week post-TAM in Fig. 3B (day 0 in vitro = 1-week post-TAM) as they constitute the starting point for both of those in vitro and in vivo experiments, respectively. Means and standard deviations are indicated. **p < 0.01, *p < 0.05. Number of replicates-**D** WT *n* = 5; from 5 different experiments. Low induction: TAM-*HSC-Scl-CRE-ERT*^*+/CRE*^*;NOTCH3*^*+/C455R*^*;Dnmt3a*^*+/+*^ t0 *n* = 6, t6 *n* = 6, t15 *n* = 4, t21 *n* = 5 from 4 independent experiments; TAM-*HSC-Scl-CRE-ERT*^*+/CRE*^;*NOTCH3*^*C455R/C455R*^*;Dnmt3a*^*+/+*^ t0 *n* = 8, t6 *n* = 8, t15 *n* = 6, t21 *n* = 7 from 6 independent experiments; TAM-*HSC-Scl-CRE-ERT*^*+/CRE*^*;NOTCH3*^*+/C455R*^*;Dnmt3a*^*+/R878H*^ t0 *n* = 9, t6 *n* = 9, t15 *n* = 9, t21 *n* = 8 from 7 independent experiments; TAM-*HSC-Scl-CRE-ERT*^*+/CRE*^;*NOTCH3*^*C455R/C455R*^*;Dnmt3a*^*+/R878H*^ t0 *n* = 5, t6 *n* = 5, t15 *n* = 5, t21 *n* = 4 from 3 independent experiments. High induction: TAM-*HSC-Scl-CRE-ERT*^*+/CRE*^*;NOTCH3*^*+/C455R*^*;Dnmt3a*^*+/+*^ t0 *n* = 5, t6 *n* = 4, t15 *n* = 5, t21 *n* = 5 from 3 independent experiments; TAM-*HSC-Scl-CRE-ERT*^*+/CRE*^;*NOTCH3*^*C455R/C455R*^*;Dnmt3a*^*+/+*^ t0 *n* = 12, t6 *n* = 12, t15 *n* = 12, t21 *n* = 11 from 9 independent experiments; TAM-*HSC-Scl-CRE-ERT*^*+/CRE*^*;NOTCH3*^*+/C455R*^*;Dnmt3a*^*+/R878H*^ t0 *n* = 7, t6 *n* = 7, t15 *n* = 7, t21 *n* = 7 from 5 independent experiments; TAM-*HSC-Scl-CRE-ERT*^*+/CRE*^;*NOTCH3*^*C455R/C455R*^*;Dnmt3a*^*+/R878H*^ t0 *n* = 6, t6 *n* = 4, t15 *n* = 6, t21 *n* = 5 from 4 independent experiments. Multiple unpaired *t*-test comparisons corrected with Holm-Sidák method, parametric test. Number of replicates-**E** Low induction: TAM-*HSC-Scl-CRE-ERT*^*+/CRE*^*;Dnmt3a*^*+/R878H*^ t0 *n* = 5; t21 = 3 from 4 independent experiments; TAM-*HSC-Scl-CRE-ERT*^*+/CRE*^*;NOTCH3*^*+/C455R*^*;Dnmt3a*^*+/R878H*^ GFP− t0 *n* = 3; t21 *n* = 4; GFP+ t0 *n* = 4; t21 *n* = 3 from 3 independent experiments (for both GFP− and GFP+); TAM-*HSC-Scl-CRE-ERT*^*+/CRE*^;*NOTCH3*^*C455R/C455R*^*;Dnmt3a*^*+/R878H*^ GFP− t0 *n* = 3; t21 *n* = 3; GFP+ t0 *n* = 3, t21 *n* = 3 from 3 independent experiments (for both GFP− and GFP+). High induction: TAM-*HSC-Scl-CRE-ERT*^*+/CRE*^*;Dnmt3a*^*+/R878H*^ t0 *n* = 5; t21 = 4 from 4 independent experiments; TAM-*HSC-Scl-CRE-ERT*^*+/CRE*^*;NOTCH3*^*+/C455R*^*;Dnmt3a*^*+/R878H*^ GFP− t0 *n* = 7; t21 *n* = 3; GFP+ t0 *n* = 8; t21 *n* = 4 from 4 independent experiments; TAM-*HSC-Scl-CRE-ERT*^*+/CRE*^;*NOTCH3*^*C455R/C455R*^*;Dnmt3a*^*+/R878H*^ GFP− t0 *n* = 7; t21 *n* = 6; GFP+ t0 *n* = 6, t21 *n* = 5 from 5 independent experiments. Parametric unpaired two-tailed *t*-test. Source data in Supplementary Table 4.

To determine the ability of CADASIL-related *NOTCH3* mutations to promote CH, we focused on the *NOTCH*^*C455R*^ mutation, detected in CADASIL patients with early stroke episodes [26], and employed a CRE-inducible *NOTCH3*^*fl-C455R*^ allele. In this mouse, the human *NOTCH3*^*C455R*^ cDNA and GFP (separated by an internal ribosome entry site sequence, IRES) are knocked into the mouse *Rosa26* locus (*R26*) [20] (Fig. 1Ai). A floxed transcriptional STOP cassette blocks the expression of *NOTCH3*^*C455R*^ cDNA and GFP and can be excised by CRE activity (Fig. 1Ai). Here, GFP expression enables tracking of NOTCH3-C455R-expressing cells. The *HSC-Scl-CRE-ERT* allele [25] (with expression confined to HSPCs) allows TAM-inducible expression of NOTCH3-C455R and GFP restricted to HSPCs (Fig. 1Aii).

To induce *Dnmt3a*^*R878H*^ mutation in HSPCs we combined a CRE-inducible *Dnmt3a*^*R878H*^ mouse strain [34] with the *HSC-Scl-CRE-ERT* allele and generated *HSC-Scl-CRE-ERT*^*+/CRE*^*; Dnmt3a*^*+/R878H*^ mice (Fig. 1B). To evaluate the effect of *NOTCH3*^*C455R*^ and *Dnmt3a*^*R878H*^ in HSPCs, we TAM-treated 7–8-week-old *HSC-Scl-CRE-ERT*^*+/CRE*^*;NOTCH3*^*C455R/+*^ (shorthanded as *+/CRE; NOTCH3*^*C455R/+*^) and *HSC-Scl-CRE-ERT*^*+/CRE*^*;NOTCH3*^*C455R/C455R*^ mice in *Dnmt3a*^*+/R878H*^ and *Dnmt3a*^*+/+*^ backgrounds (Fig. 1C). Two TAM regimens were employed to render different percentages of mutated cells: low-induction (a single dose of 1 mg of TAM) and high-induction (five consecutive doses of 2 mg of TAM per day). HSPCs (i.e. Lineage^−^Sca-1^+^c-Kit^+^, LSK cells) were isolated 1 week post-TAM and cultured in expansion media [32] for 21 days to study the competition between non-mutant cells and *NOTCH3*^*C455R*^ and *Dnmt3a*^*R878H*^ single- and double-mutant cells ex vivo (Fig. 1C). We tracked the accumulation of *NOTCH3*^*C455R*^-GFP^+^ cells by flow cytometry and changes in the frequency of *Dnmt3a*^*R878H*^ HSPCs over time by PCR analysis. Particularly, GFP^+^ and GFP^−^ HSPCs were sorted from the cell cultures at day 0 and day 21 post-culture. Sorted HSPCs were plated into semi-solid media to allow clonal expansion to facilitate genotyping. Individual colonies were then genotyped for *Dnmt3a*^*R878H*^ (Fig. 1C and Supplementary Fig. 2C).

*NOTCH3*^*C455R*^ GFP^+^ cells (both *NOTCH3*^*+/C455R*^ and *NOTCH3*^*C455R/C455R*^) progressively accumulated ex vivo in expansion media following low- and high-induction TAM treatments (Fig. 1D). *Dnmt3a*^*R878H*^ expression enhanced the accumulation of GFP^+^ *NOTCH3*^*C455R/C455R*^ cells (Fig. 1D). This was statistically significant following low induction (Fig. 1D). Low-induction treatment allowed higher resolution for GFP accumulation as the initial percentages of GFP^+^ cells were lower (~10%). Analysis of the accumulation of *Dnmt3a*^*R878H*^ mutant cells by genotyping of clonally expanded cells from sorted *NOTCH3*^*C455R*^ GFP^+^ and GFP^−^ cells showed that *Dnmt3a*^*R878H*^ mutant HSPCs tend to accumulate over time regardless of *NOTCH3* mutational status, but this trend was only statistically significant for GFP^+^ *Dnmt3a*^*R878H*^ mutant cells in a *NOTCH3*^*C455R/C455R*^ background under high induction (Fig. 1E and Supplementary Fig. 2C).

Overall, these data showed that *NOTCH3*^*C455R*^ expression confers a fitness advantage to HSPCs ex vivo. This advantage was further enhanced by the co-expression of *Dnmt3a*^*R878H*^ mutation supporting a functional cooperation among both mutations.

### *NOTCH3*^*C455R*^ HSPCs expand in vivo

**Fig. 2:**
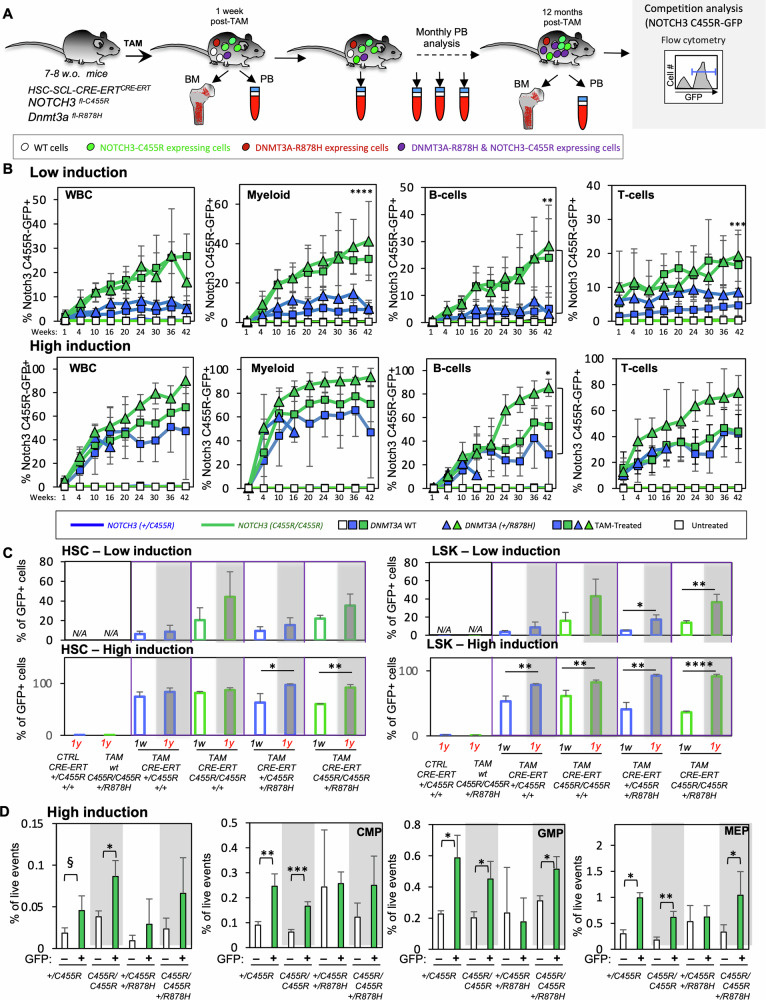
NOTCH3-C455R expression confers in vivo a fitness advantage to *NOTCH3*^*C455R*^ carrier HSPCs. **A** Experimental approach. *HSC-Scl-CRE-ERT*^*+/CRE*^*;NOTCH3*^*C455R/+*^ (shorthanded as *+/CRE;NOTCH3*^*C455R/+*^) and *+/CRE;NOTCH3*^*C455R/C455R*^ mice in a *Dnmt3a*^*+/R878H*^ and *Dnmt3a*^*+/+*^ background were TAM-treated to induce NOTCH3-C455R and DNMT3A-R878H expression in HSPCs in vivo. The accumulation of NOTCH3-C455R-expressing cells was tracked over time via flow cytometry (i.e. GFP^+^ cells) in the peripheral blood (PB) and bone marrow (BM). **B** Two TAM-treatments (low and high-TAM) were employed to induce different initial numbers of *NOTCH3*^*C455R*^ and *DNMT3A*^*R878H*^ mutant cells. NOTCH3-C455R expressing white blood cells (WBCs) including myeloid, B- and T-lymphoid cells accumulated over time. Evolution on the % of NOTCH3-C455R-GFP^+^ cells is shown for myeloid, B-cells and T-cells. TAM-treated *+/CRE;NOTCH3*^*C455R/C455R*^ accumulated faster and the presence of *Dnmt3a*^*R878H*^ accelerated the expansion of double mutant *+/CRE;NOTCH3*^*C455R/C455R*^*; Dnmt3a*^*+/R878H*^ B-cells at high induction. **C** Changes in the % of NOTCH3-C455R-GFP^+^ cells are shown for HSCs and Lin^−^Sca-1^+^c-Kit^+^ (LSK) HSPCs at 1-week and 1-year post-TAM. NOTCH3-C455R-GFP^+^ HSCs and HSPCs accumulated over time (see also Supplementary Fig. 3). **D** Analysis of the cellular composition of GFP^+^ *NOTCH3*^*C455R*^ and GFP^−^ control HSPCs in TAM-treated *+/CRE;NOTCH3*^*C455R/+*^ and *+/CRE;NOTCH3*^*C455R/C455R*^ mice in *Dnmt3a*^*+/R878H*^ and *Dnmt3a*^*+/+*^ backgrounds 1-year post-TAM. HSPCs were analyzed for the presence of HSC, MPP, HPC-1&2, CMP, GMP, MEP and CLP (see also Supplementary Fig. 5). HSCs, GMPs, CMPs and MEPs were enriched within *NOTCH3*^*C455R*^ GFP^+^ HSPCs. Results are shown for high induction. Means and standard deviations are indicated. ****p < 0.001, ***p < 0.001, **p < 0.01, *p < 0.05, ^§^*p* < 0.1. Number of replicates-**B** Untreated-*HSC-Scl-CRE-ERT*^*+/CRE*^*;NOTCH3*^*+/C455R*^*;Dnmt3a*^*+/+*^ t1, t4, t10, t16, t20, t24, t30, t36 *n* = 5, t42 *n* = 4; untreated-*HSC-Scl-CRE-ERT*^*+/CRE*^*;NOTCH3*^*C455R/C455R*^*;Dnmt3a*^*+/+*^ t1, t4, t10, t16, t20, t24, t30, t42 *n* = 3, t36 *n* = 2. Low induction: TAM-*HSC-Scl-CRE-ERT*^*+/CRE*^*;NOTCH3*^*+/C455R*^*;Dnmt3a*^*+/+*^ t1 and t4 *n* = 11 and t10, t16, t20, t24, t30, t36, t42 *n* = 8 for WBC, B cells and myeloid. For T cells t1, t10, t16, t20, t24, t30, t36 *n* = 8 and t4 *n* = 10. TAM-*HSC-Scl-CRE-ERT*^*+/CRE*^*;NOTCH3*^*C455R/C455R*^*;Dnmt3a*^*+/+*^ t1 and t4 *n* = 11, t10, t30 *n* = 8, t16, t24, t36 *n* = 7, t20 *n* = 6, t42 *n* = 5. TAM-*HSC-Scl-CRE-ERT*^*+/CRE*^*; NOTCH3*^*C455R/C455R*^*;Dnmt3a*^*+/R878H*^ t1, t4, t10, t16, t20, t24, t30, t36, t42 *n* = 5. TAM-*HSC-Scl-CRE-ERT*^*+/CRE*^*;NOTCH3*^*+/C455R*^*;Dnmt3a*^*+/R878H*^ t1, t4 *n* = 5, t10 *n* = 4, t16, t20, t24, t30, t36 *n* = 3, t42 *n* = 2 for WBC. For B cells, myeloid and T cells t1, t16, t20, t24, t30, t36 *n* = 3, t4 *n* = 5, t10 *n* = 4 and t42 *n* = 2. Low induction: TAM-*HSC-Scl-CRE-ERT*^*+/CRE*^*;NOTCH3*^*+/C455R*^*;Dnmt3a*^*+/+*^ t1 *n* = 12, t4 *n* = 9, t10 *n* = 7, t16, t20, t24, t30 *n* = 8, t36 *n* = 6 and t42 *n* = 2 for WBC. For B cells and myeloid t1 *n* = 12, t4 *n* = 9, t10 *n* = 8, t16, t20, t24, t30 and t36 *n* = 6 and t42 *n* = 3. For T cells t1 *n* = 5, t4 *n* = 9, t10 *n* = 7, t16, t24, t30 and t36 *n* = 6, t20 *n* = 5, t42 *n* = 3. TAM-*HSC-Scl-CRE-ERT*^*+/CRE*^*;NOTCH3*^*C455R/C455R*^*;Dnmt3a*^*+/+*^ t1, t4 *n* = 14, t10 *n* = 12, t16, t20, t24, t30 and t36 *n* = 11 and t42 *n* = 9 for WBC, B cells, myeloid and T cells. TAM-*HSC-Scl-CRE-ERT*^*+/CRE*^*;NOTCH3*^*+/C455R*^*;Dnmt3a*^*+/R878H*^ t1, t4 and t10 *n* = 7, t16 *n* = 6, t20 *n* = 5, t24, t30, t36 and t42 *n* = 3 for WBC. For B cells, myeloid and T cells t1, t4 and t10 *n* = 7, t16 and t20 *n* = 6, t24 and t30 *n* = 4, t36 and t42 *n* = 3. TAM-*HSC-Scl-CRE-ERT*^*+/CRE*^*;NOTCH3*^*+/C455R*^*;Dnmt3a*^*+/R878H*^ t1 *n* = 5, t4 and t10 *n* = 2 and t16 *n* = 1 for WBC, B cells, myeloid and T cells. Parametric unpaired two-tailed *t*-test. Number of replicates-**C** Untreated-*HSC-Scl-CRE-ERT*^*+/CRE*^*;NOTCH3*^*+/C455R*^*;Dnmt3a*^*+/+*^ *n* = 4. One-week, low induction. TAM-*HSC-Scl-CRE-ERT*^*+/CRE*^*;NOTCH3*^*+/C455R*^*;Dnmt3a*^*+/+*^ *n* = 5, TAM-*HSC-Scl-CRE-ERT*^*+/CRE*^*;NOTCH3*^*+/C455R*^*;Dnmt3a*^*+/R878H*^ *n* = 3, TAM-*HSC-Scl-CRE-ERT*^*+/CRE*^*;NOTCH3*^*C455R/C455R*^*;Dnmt3a*^*+/+*^ *n* = 3, TAM-*HSC-Scl-CRE-ERT*^*+/CRE*^*;NOTCH3*^*C455R/C455R*^*;Dnmt3a*^*+/R878H*^ TAM *n* = 3. One week, high induction. TAM-*HSC-Scl-CRE-ERT*^*+/CRE*^*;NOTCH3*^*+/C455R*^*;Dnmt3a*^*+/+*^ *n* = 5, TAM-*HSC-Scl-CRE-ERT*^*+/CRE*^*;NOTCH3*^*+/C455R*^*;Dnmt3a*^*+/R878H*^ *n* = 3, TAM-*HSC-Scl-CRE-ERT*^*+/CRE*^*;NOTCH3*^*C455R/C455R*^*;Dnmt3a*^*+/+*^ *n* = 5, TAM-*HSC-Scl-CRE-ERT*^*+/CRE*^*;NOTCH3*^*C455R/C455R*^*;Dnmt3a*^*+/R878H*^ *n* = 3. One year, low induction. TAM-*HSC-Scl-CRE-ERT*^*+/CRE*^*;NOTCH3*^*+/C455R*^*;Dnmt3a*^*+/+*^ *n* = 6, TAM-*HSC-Scl-CRE-ERT*^*+/CRE*^*;NOTCH3*^*+/C455R*^*;Dnmt3a*^*+/R878H*^ *n* = 3, TAM-*HSC-Scl-CRE-ERT*^*+/CRE*^*;NOTCH3*^*C455R/C455R*^*;Dnmt3a*^*+/+*^ *n* = 3, TAM-*HSC-Scl-CRE-ERT*^*+/CRE*^*;NOTCH3*^*C455R/C455R*^*;Dnmt3a*^*+/R878H*^ *n* = 5. One year, high induction. TAM-*HSC-Scl-CRE-ERT*^*+/CRE*^*;NOTCH3*^*+/C455R*^*;Dnmt3a*^*+/+*^ *n* = 3, TAM-*HSC-Scl-CRE-ERT*^*+/CRE*^*;NOTCH3*^*+/C455R*^*;Dnmt3a*^*+/R878H*^ *n* = 2, TAM-*HSC-Scl-CRE-ERT*^*+/CRE*^*;NOTCH3*^*C455R/C455R*^*;Dnmt3a*^*+/+*^ *n* = 3, TAM-*HSC-Scl-CRE-ERT*^*+/CRE*^*; NOTCH3*^*C455R/C455R*^*; Dnmt3a*^*+/R878H*^ *n* = 3; TAM-*HSC-Scl-CRE-ERT*^*+/+*^*;NOTCH3*^*C455R/C455R*^*;Dnmt3a*^*+/R878H*^ *n* = 3. Parametric unpaired two-tailed *t*-test. Number of replicates-**D** One year, high induction. TAM-*HSC-Scl-CRE-ERT*^*+/CRE*^*;NOTCH3*^*+/C455R*^*;Dnmt3a*^*+/+*^ *n* = 3; TAM-*HSC-Scl-CRE-ERT*^*+/CRE*^*;NOTCH3*^*C455R/C455R*^*;Dnmt3a*^*+/+*^ *n* = 3; TAM-*HSC-Scl-CRE-ERT*^*+/CRE*^*;NOTCH3*^*+/C455R*^*;Dnmt3a*^*+/R878H*^ *n* = 3; TAM-*HSC-Scl-CRE-ERT*^*+/CRE*^*;NOTCH3*^*C455R/C455R*^*;Dnmt3a*^*+/R878H*^ *n* = 3. Parametric unpaired two-tailed *t*-test. Source data in Supplementary Table 4.

To determine if *NOTCH3*^*C455R*^ increases HSPC fitness in vivo, we TAM-treated (under low- and high-induction TAM regimes) *+/CRE;NOTCH3*^*C455R/+*^ and *+/CRE;NOTCH3*^*C455R/C455R*^ mice in a *Dnmt3a*^*+/R878H*^ and *Dnmt3a*^*+/+*^ backgrounds (Fig. 2A). The frequency of *NOTCH3*^*C455R*^-GFP^+^ cells was tracked over time both in the PB and BM by flow cytometry at 1 week and 12 months post-TAM (Fig. 2B, C and Supplementary Fig. 3). Serial bleeding showed that *NOTCH3*^*C455R*^-GFP^+^ (both *NOTCH3*^*+/C455R*^ and *NOTCH3*^*C455R/C455R*^) white blood cells (WBCs), including myeloid and B- and T- lymphoid cells, accumulated over time (Fig. 2B). Faster accumulation of GFP^+^ cells was observed in *NOTCH3*^*C455R/C455R*^ compared to *NOTCH3*^*+/C455R*^ mice, revealing a *NOTCH3*^*C455R*^-dose effect (Fig. 2B). *Dnmt3a*^*R878H*^ further enhanced the accumulation of GFP^+^ cells, although this was only statistically significant for B-cells following high induction, where variances among conditions were smaller (Fig. 2B).

Importantly, analysis of HSPC BM compartments [including HSC, HPC-1, HPC-2, MPPs, common lymphoid progenitors (CLP), common myeloid progenitors (CMP), megakaryocyte-erythrocyte progenitor (MEP) and granulocyte-monocyte progenitor (GMP)] of TAM-treated mice 1 week- and 1 year- post-treatment revealed that *NOTCH3*^*C455R*^-GFP^+^ HSPCs accumulated over time in all HSPC compartments and genotypes (Fig. 2C and Supplementary Fig. 3). Mice harboring *NOTCH3*^*C455R*^ and/or *Dnmt3a*^*R878H*^ did not exhibit major alterations of cellular and biochemical blood parameters (Supplementary Fig. 4). Yet, total WBC were increased in *+/CRE;NOTCH3*^*C455R/+*^*Dnmt3a*^*+/R878H*^ mice.

Further, to characterize the differentiation ability of *NOTCH3*^*C455R*^ HSPCs, we performed phenotypic analyses on the distribution of HSPC populations within GFP^+^ and GFP^−^ cells in the BM of TAM-treated *+/CRE;NOTCH3*^*C455R/+*^ (*NOTCH3*^*C455R/+*^) and *+/CRE;NOTCH3*^*C455R/C455R*^ (*NOTCH3*^*C455R/C455R*^) mice in *Dnmt3a*^*+/R878H*^ and *Dnmt3a*^*+/+*^ backgrounds 1 year post-treatment (Fig. 2D and Supplementary Fig. 5). Here, the frequency of HSCs was significantly enriched within GFP^+^ *NOTCH3*^*C455R/C455R*^ HSPCs compared to the frequency of HSCs among GFP^−^ *NOTCH3*^*C455R/C455R*^ cells (under high-induction) (Fig. 2D). The frequency of HSCs trended up but did not reach statistical significance (*p* < 0.1) within GFP^+^ *NOTCH3*^*C455R/+*^ HSPCs compared to the frequency of HSC within GFP^−^ *NOTCH3*^*C455R/+*^ HSPC (Fig. 2D). Similarly, CMPs, GMPs and MEPs were significantly enriched within *NOTCH3*^*C455R*^-GFP^+^ cells in all analyzed genotypes but the *+/CRE;NOTCH3*^*C455R/+*^*Dnmt3a*^*+/R878H*^ cohort (Fig. 2D). These accumulations were not commiserated with a differentiation blockage as *NOTCH3*^*C455R*^ HSPCs and differentiated mature blood *NOTCH3*^*C455R*^ mutant cells progressively accumulated in the PB over time post-TAM (Fig. 2B).

### *NOTCH3*^*C455R*^ mutant HSPCs expand more rapidly

**Fig. 3:**
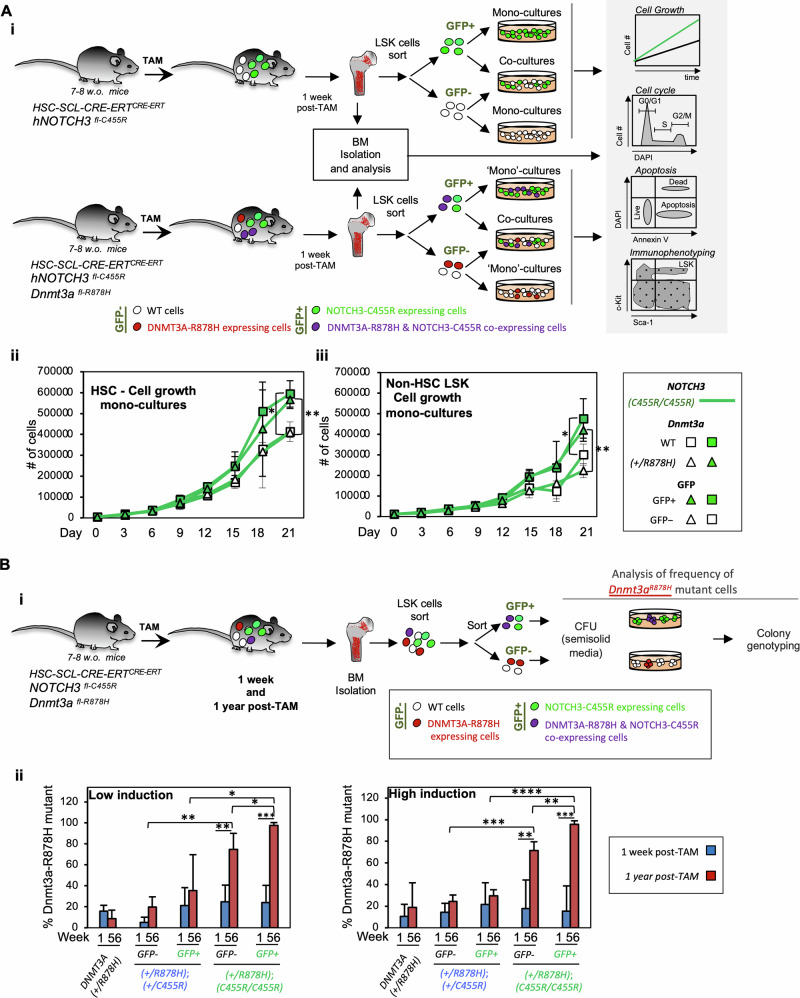
*NOTCH3*^*C455R*^ expression results in faster proliferation of HSCs and supports the expansion of *Dnmt3a*^*R878H*^*HSPCs in a non-cell autonomous fashion.* **A** BM-HSPCs from TAM-treated *HSC-Scl-CRE-ERT*^*+/CRE*^*;NOTCH3*^*C455R/C455R*^ mice in a *Dnmt3a*^*+/R878H*^ and *Dnmt3a*^*+/+*^ background were grown as mono-cultures and co-cultures of *NOTCH3*^*C455R*^-GFP^+^ HSPCs and *Ctrl*-GFP^-^ HSPCs. **Ai** Experimental schematic. As mono-cultures, *NOTCH3*^*C455R*^-GFP^+^ cells grew faster than GFP^−^ internal control cells. Cell growth curves are shown for HSCs (**Aii**) and non-HSC-LSK cells (**Aiii**). Freshly isolated BM and in vitro co-cultures and mono-cultures of isolated HSPCs were analyzed by flow cytometry for cell cycle distribution via Ki67/DAPI staining and apoptosis (AnnexinV/DAPI staining) in Supplementary Figs. 6, 7. **B** Quantification on the in vivo selection of *DNMT3A*^*R878H*^ cells. Genotyping of clonally expanded GFP^+^ and GFP^−^ sorted LSK cells 1 week and 1-year post-TAM revealed that GFP^+^ *Dnmt3a*^*R878H*^ and GFP^−^ *Dnmt3a*^*R878H*^ mutant cells accumulated in vivo under the presence of GFP^+^ *HSC-Scl-CRE-ERT*^*+/CRE*^*;NOTCH3*^*C455R/C455R*^*;Dnmt3a*^*+/R878H*^ cells. GFP^+^ *Dnmt3a*^*R878H*^ accumulated more dramatically than GFP^−^ *Dnmt3a*^*R878H*^ HSPCs. Data at 1 week post-TAM is the same as day 0 in Fig. 1E (1 week = day 0) as they constitute the starting point for those in vivo and in vitro experiments. **A**, **B** Means and standard deviations are indicated. ****p < 0.001, ***p < 0.001, **p < 0.01, *p < 0.05. Parametric unpaired two-tailed *t*-test. Source data in Supplementary Table 4. Number of replicates-**Aii** From three independent experiments, TAM-*HSC-Scl-CRE-ERT*^*+/CRE*^*;NOTCH3*^*C455R/C455R*^*;Dnmt3a*^*+/+*^ *n* = 3 TAM-*HSC-Scl-CRE-ERT*^*+/CRE*^*;NOTCH3*^*C455R/C455R*^*;Dnmt3a*^*+/R878H*^ *n* = 3. Number of replicates-**Aiii** From three independent experiments TAM-*HSC-Scl-CRE-ERT*^*+/CRE*^*;NOTCH3*^*C455R/C455R*^*;Dnmt3a*^*+/+*^ *n* = 3, TAM-*HSC-Scl-CRE-ERT*^*+/CRE*^*;NOTCH3*^*C455R/C455R*^*;Dnmt3a*^*+/R878H*^ *n* = 3. Number of replicates-**Bii** Low induction: TAM-*HSC-Scl-CRE-ERT*^*+/CRE*^*; Dnmt3a*^*+/R878H*^ t1 *n* = 5; t56 *n* = 4 from 4 independent experiments; TAM-*HSC-Scl-CRE-ERT*^*+/CRE*^*;NOTCH3*^*+/C455R*^*;Dnmt3a*^*+/R878H*^ GFP^−^ t1 *n* = 3; t56 *n* = 3; GFP^+^ t1 *n* = 4; t56 *n* = 3 from 3 independent experiments; TAM-*HSC-Scl-CRE-ERT*^*+/CRE*^*;NOTCH3*^*C455R/C455R*^*;Dnmt3a*^*+/R878H*^ GFP^−^ t1 *n* = 3; t56 *n* = 4; GFP^+^ t1 *n* = 3, t56 *n* = 34 from 3 independent experiments. High induction: TAM-*HSC-Scl-CRE-ERT*^*+/CRE*^*; Dnmt3a*^*+/R878H*^ t1 *n* = 5; t56 = 5 from 4 independent experiments; TAM-*HSC-Scl-CRE-ERT*^*+/CRE*^*;NOTCH3*^*+/C455R*^*;Dnmt3a*^*+/R878H*^ GFP^−^ t1 *n* = 7; t56 *n* = 3; GFP^+^ t1 *n* = 8; t56 *n* = 3 from 4 independent experiments; TAM-*HSC-Scl-CRE-ERT*^*+/CRE*^*;NOTCH3*^*C455R/C455R*^*;Dnmt3a*^*+/R878H*^ GFP^−^ t1 *n* = 7; t56 *n* = 4; GFP^+^ t1 *n* = 6, t21 *n* = 4 from 5 independent experiments. Parametric unpaired two-tailed *t*-test.

To determine the cellular mechanisms that confer a selective advantage to *NOTCH3*^*C455R*^ HSPCs resulting in their expansion over time (as shown for HSC and LSK cells), we TAM-treated *+/CRE;NOTCH3*^*C455R/C455R*^ mice in *Dnmt3a*^*+/R878H*^ and *Dnmt3a*^*+/+*^ backgrounds (three doses of 2 mg TAM in consecutive days) (Fig. 3A and Supplementary Fig. 6). One-week post-TAM, BM HSPCs (i.e. HSC, HPC-1, HPC-2, MPPs, CLP, CMP, MEP and GMP) were analyzed for apoptosis and cell cycle status (Supplementary Fig. 6). We detected no significant differences in the distribution of cell cycle phases and apoptosis levels for any *NOTCH3*^*C455R*^GFP^+^ HSPCs compared to their GFP^−^ control cells (Supplementary Fig. 6).

The fitness advantage of *NOTCH3*^*C455R*^ HPSCs could also be due to non-cell autonomous effects of *NOTCH3*^*C455R*^ HPSCs over wt HSPCs by inducing their apoptosis, proliferation and/or differentiation. To unveil this, GFP^+^ and GFP^−^ LSK cells were sorted from TAM-treated *+/CRE;NOTCH3*^*C455R/C455R*^ mice in *Dnmt3a*^*+/R878H*^ and *Dnmt3a*^*+/+*^ backgrounds. GFP^+^ and GFP^−^ LSK cells were plated either as mono-cultures or co-cultures of GFP^+^/GFP^−^ cells in expansion media (Fig. 3A and Supplementary Fig. 7). Here, we observed no changes in the cell cycle status, apoptosis, and differentiation potential of GFP^−^ cells cultured with or without *NOTCH3*^*C455R*^ GFP^+^ cells (Supplementary Fig. 7). Thus, *NOTCH3*^*C455R*^ HSPCs are not detrimental to wild type neighboring cells ex vivo.

We next assessed if *NOTCH3*^*C455R*^ expression perturbed the growth of GFP^+^ and GFP^−^ HSPCs. Here, *NOTCH3*^*C455R*^-GFP^+^ or GFP^−^ HSCs and HSPCs (non-HSC LSK cells) were cultured separately, revealing that *NOTCH3*^*C455R*^-GFP^+^ HSCs and HSPCs cells expanded more rapidly than control cells (Fig. 3Aii, iii). The presence of *Dnmt3a*^*R878H*^ did not provide any additional cell autonomous growth advantage (Fig. 3Aii, iii).

Overall, these analyses indicate that the fitness advantage driven by *NOTCH3*^*C455R*^ expression in HSCs is cell intrinsic and based on faster cell proliferation.

### *NOTCH3*^*C455R*^ HSPCs confer a non-cell autonomous advantage to *DNMT3A*^*R878H*^ HSPCs

Analysis of the prevalence of *Dnmt3a*^*R878H*^ via colony genotyping of clonally expanded *NOTCH3*^*C455R*^-GFP^+^ and GFP^−^ LSK cells isolated from the BM of TAM-treated mice 1-week and 1-year post-TAM showed that both GFP^−^ *NOTCH3*^*C455R/C455R*^ and GFP^+^ *NOTCH3*^*C455R/C455R*^ HSPCs harboring *Dnmt3a*^*R878H*^ mutation exhibit a selective advantage and expand in vivo ~4-fold and ~5-fold times, respectively, when compared to their initial prevalence at low and high induction (Fig. 3B). This dramatic increase was not detected in *NOTCH3*^*+/C455R*^ or *NOTCH3*^*+/+*^ HSPCs, indicating a *NOTCH3*^*C455R*^ dose-dependent effect.

To evaluate the molecular interaction among *NOTCH3*^*C455R*^ and *Dnmt3a*^*R878H*^ mutations we isolated HSCs, MPPs, HPC-1 and HPC-2 cells from TAM-treated *HSC-Scl-CRE-ERT* ^*+/CRE*^*;NOTCH3*^*C455R/C455R*^ mice in *Dnmt3a*^*+/R878H*^ and *Dnmt3a*^*+/+*^ backgrounds and *HSC-Scl-CRE-ERT*^*+/CRE*^*;Dnmt3a*^*+/R878H*^ mice. As controls we also obtained same HSPC populations from TAM-treated *HSC-Scl-CRE-ERT*^*+/CRE*^;*NOTCH3*^*+/+*^*;Dnmt3a*^*+/+*^, *HSC-Scl-CRE-ERT*^*+/+*^*;NOTCH3*^*C455R/C455R*^ and *HSC-Scl-CRE-ERT*^*+/+*^*;NOTCH3*^*C455R/C455R*^*;Dnmt3a*^*+/R878H*^ mice (Supplementary Fig. 9). By qRT-PCR we analyzed at the transcriptional level genes previously described to be perturbed in a *Dnmt3a*^*−/−*^ background [36] as a “*Dnmt3a*-mutant-signature”. DNMT3A-R878H expression triggered the upregulation of many of these “*Dnmt3a*-mutant-signature” genes in all the progenitors, although these differences were only statistically significant for *Foxo1*, *Gata3*, *Vasn*, *Pu.1* and *Mef2c* in some HSPCs (Supplementary Fig. 9). Notably, *NOTCH3*^*C455R*^ and *Dnmt3a*^*R878H*^ co-expression reverted all these phenotypes. The analysis of molecules directly implicated in cell cycle progression showed that *Dnmt3a*^*R878H*^ mutant MPPs significantly upregulated *Ccne1*, critical in G1-S transition. Interestingly, *NOTCH3*^*C455R*^ and *Dnmt3a*^*R878H*^ co-expression resulted in the downregulation of CIP/KIP cell cycle inhibitors (*p21*, *p27* and *p57*) in MPPs (Supplementary Fig. 9B), which can likely facilitate cell cycle progression in double mutant HSPCs. Although not statistically significant this trend was also observed in HSCs where *NOTCH3*^*C455R*^ and *Dnmt3a*^*R878H*^ co-expression also reverted the upregulation of CIP/KIP cell cycle inhibitors observed in *Dnmt3a*^*R878H*^ HSCs (Supplementary Fig. 9A).

Very intriguingly, GFP^−^*Dnmt3a*^*R878H*^ LSK cells (which do not express *NOTCH3*^*C455R*^) only significantly expanded in the presence of GFP^+^*NOTCH3*^*C455R/C455R*^ hematopoietic cells and almost at the same level as GFP^+^*Dnmt3a*^*R878H*^*NOTCH3*^*C455R/C455R*^ (Fig. 3B). This was consistently detected both at low and high-induction regimes in vivo (Fig. 3B). Importantly, this demonstrates a non-cell autonomous positive effect of *NOTCH3*^*C455R/C455R*^ cells on the expansion of *Dnmt3a*^*R878H*^ mutant cells. Very intriguingly, this suggests that CADASIL patients (at least those carrying *NOTCH3*^*C455R*^ germline mutations) would be much more prone to the development of *DNMT3A*^*R882H*^ CH than the general population. Additionally, *Dnmt3a*^*R878H*^ mutant cells significantly accumulated more in GFP^+^*Dnmt3a*^*R878H*^ than in GFP^−^*Dnmt3a*^*R878H*^ indicating a molecular cooperation among *NOTCH3*^*C455R*^ and *Dnmt3a*^*R878H*^ mutations.

### Widespread NOTCH3-C455R expression in the hematopoietic system resets clonal competition to a new basal homeostatic level

**Fig. 4:**
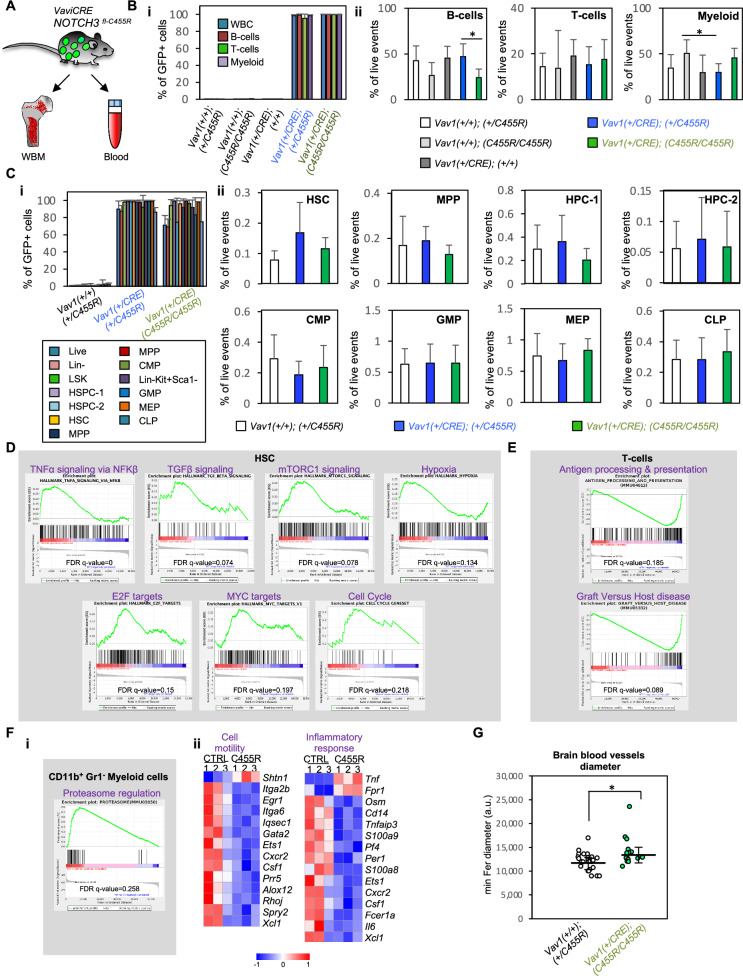
Widespread expression of NOTCH3-C455R in the hematopoietic system reinstates clonal competition homeostasis but perturbs the transcriptional profile of HSCs, T-cells and myeloid cells. **A** *Vav1CRE* leads to CRE expression in hematopoietic tissues from day E12.5 of embryonic development, and hence to broad hematopoietic recombination of floxed alleles. **B** The hematopoietic system of *Vav1CRE*^*+/CRE*^;*NOTCH3*^*C455R/+*^ and *Vav1CRE*^*+/CRE*^*;NOTCH3*^*C455R/C455R*^ mice (both referred globally as *Vav1*^*CRE*^ *NOTCH3*^*C455R*^ mice) was analyzed. **Bi** PB shows almost 100% of NOTCH3-C455R-GFP^+^ cells**. Bii** No differences were observed for the distribution of myeloid and lymphoid cells in the PB of *Vav1*^*CRE*^*NOTCH3*^*C455R*^ mice. **C** Similarly WBM shows almost 100% of NOTCH3-C455R-GFP^+^ cells (**Ci**) and no differences for the distribution of HSPCs among control and *Vav1*^*CRE*^*NOTCH3*^*C455R*^ mice (**Cii**). **D**–**F** HSCs, T-cells and CD11b^+^Gr1^−^ myeloid cells were isolated from *Vav1CRE*^*+/CRE*^;*NOTCH3*^*C455R/C455R*^ mice (C455R) and *Vav1CRE*^*+/+*^*;NOTCH3*^*C455R/C455R*^ control mice (CTRL) (*n* ≥ 3). NOTCH3-C455R-expression led to the perturbation of expression of gene sets implicated in NF-κB, TGF-β and mTORC1 signaling, E2F- and MYC-target genes and cell cycle machinery in HSCs (**D**), in antigen processing and expression in T-cells (**E**) and proteasome regulation cell motility and inflammatory response in CD11b^+^Gr1^−^ myeloid cells (**F**). **D**–**F** FDR *q*-values < 0.25 were considered statistically significant. **Fii** Heatmaps show differentially expressed genes with log_2_fold-changes > 1 and <−1 and *p*-adj < 0.05. **G** Blood vessels in the brain parenchyma of *Vav1CRE*^*+/CRE*^*;NOTCH3*^*C455R/C455R*^ (*n* = 5) and *NOTCH3*^*C455R/C455R*^ mice (*n* = 8) three typical cross-sections were analyzed and captured from similar areas per mouse and Feret’s diameters evaluated. Minimum Feret’s diameters for each cohort are shown. Each dot represents an independent blood vessel. Hematoxylin & eosin staining of the analyzed blood vessels shown in Supplementary Fig. 10. **B**, **C**, **G** Means and standard deviations are indicated. Parametric unpaired two-tailed *t*-test. *p < 0.05. Source data in Supplementary Table 4. Number of replicates-**Bi**, **ii** *Vav1CRE*^*+/CRE*^*;NOTCH3*^*C455R/C455R*^ *n* = 4; *Vav1CRE*^*+/CRE*^*;NOTCH3*^*+/C455R*^ *n* = 18; *Vav1CRE*^*+/CRE*^*;NOTCH3*^*+/+*^ *n* = 5; *Vav1CRE*^*+/+*^*;NOTCH3*^*C455R/C455R*^ *n* = 4; *Vav1CRE*^*+/+*^*;NOTCH3*^*+/C455R*^ *n* = 10. Parametric unpaired two-tailed *t*-test. Number of replicates-**Ci**, **ii** *Vav1CRE*^*+/CRE*^*;NOTCH3*^*+/C455R*^ *n* = 5; *Vav1CRE*^*+/CRE*^*;NOTCH3*^*C455R/C455R*^ *n* = 4; *Vav1CRE*^*+/+*^*;NOTCH3*^*+/C455R*^ *n* = 9. Parametric unpaired two-tailed *t*-test. Number of replicates-**D**–**F** *Vav1CRE*^*+/+*^*;NOTCH3*^*C455R/C455R*^ *n* = 3 for CD11b^+^Gr1^−^, T-cells and B-cells, *n* = 4 for HSCs. *Vav1CRE*^*+/CRE*^*;NOTCH3*^*C455R/C455R*^ *n* = 4 for all cell types.

To generate a CADASIL-like hematopoietic system where all hematopoietic cells express *NOTCH3*^*C455R*^, we generated *Vav1CRE*^*+/CRE*^*;NOTCH3*^*C455R/+*^ and *Vav1CRE*^*+/CRE*^*;NOTCH3*^*C455R/C455R*^ mice which harbored about 100% of *NOTCH3*^*C455R*^GFP^+^ cells in their PB and BM (Fig. 4A, Bi, Ci). These mice did not show any perturbation in the distribution of cells in BM-HSPC compartments and PB cells compared to control littermates (Fig. 4Bii, Cii) and in analyzed cellular and biochemical blood parameters (Supplementary Fig. 8). Overall, these data indicate that as every cell in the hematopoietic system of these mice expressed NOTCH3-C455R, a new steady state is achieved where mutant cells display initially a similar fitness.

Transcriptional profiling of HSCs isolated from *Vav1CRE*^*+/CRE*^;*NOTCH3*^*C455R/C455R*^ and *NOTCH3*^*C455R/C455R*^ control mice by bulk mRNA sequencing and gene set enrichment analysis (GSEA) revealed that many pathways implicated in cell cycle regulation including NF-κB, TGF-β and mTORC1 signaling, E2F- and MYC-target genes and genes coding for proteins which are integral part of the cell cycle machinery were significantly enriched (FDR *q*-values < 0.25) in NOTCH3-C455R-expressing HSCs (Fig. 4D). Additionally, NOTCH3-C455R expression also resulted in the upregulation of hypoxia-related genes, key to sustain HSC function (Fig. 4D). Since NOTCH3 signaling plays a role in macrophage activation via NF-κB [37] and aberrant NOTCH signaling contributes to T-ALL growth [14], we transcriptionally profiled PB CD11b^+^Gr1^−^ myeloid cells (comprising macrophages and monocytes) and mature T-cells to investigate the effects of NOTCH3-C455R expression on these differentiated cell types. CD11b^+^Gr1^−^ and T-cells were isolated from *Vav1CRE*^*+/CRE*^*;NOTCH3*^*C455R/C455R*^ mice and *NOTCH3*^*C455R/C455R*^ control mice and transcriptionally profiled. GSEA revealed that genes implicated in antigen processing and expression were altered in T-cells (FDR *q*-values < 0.25) (Fig. 4E, F). Interestingly, NOTCH3-C455R expression marginally altered the expression levels of genes implicated in proteasome regulation in CD11b^+^Gr1^−^ cells (FDR *q*-value = 0.258) (Fig. 4F). Additionally, genes involved in cell motility and in inflammatory response were perturbed with high production of TNF-α, a master regulator of inflammation [38] (Fig. 4F).

To test if the sole presence of NOTCH3-C455R expressing hematopoietic cells could facilitate CADASIL development, we performed histopathological analysis in the brains of *Vav1CRE*^*+/CRE*^*;NOTCH3*^*C455R/C455R*^ and *NOTCH3*^*C455R/C455R*^ mice. Although mutant mice did not exhibit granular osmiophilic-like deposits or apparent vascular smooth muscle degeneration, we did observe that the vessels in the brain parenchyma of *Vav1CRE*^*+/CRE*^*;NOTCH3*^*C455R/C455R*^ mice were significantly enlarged (~17% wider) according to the obtained Feret’s diameters (Fig. 4G and Supplementary Fig. 10). This could indicate changes in blood flow regulation, vessel integrity or underlying tissue demands [39].

Overall, in a CADASIL-like hematopoietic system where every cell expresses a *NOTCH3*^*C455R*^ mutation, the global cellularity reaches a new steady state as there is no proliferative advantage driven by *NOTCH3*^*C455R*^ mutation. Yet, NOTCH3-C455R expression results in significant transcriptional perturbations in HSCs, T-cells and myeloid cells and enlarged blood vessels in the brain parenchyma.

## Discussion

CH is virtually universal in the aged population [27]. Although recent studies showed that sustained local inflammation driven by *Tet2*^*−/−*^ and *Jak2*^*V617F*^ myeloid cells facilitates the development of cardiovascular conditions [9, 10], the relevance and consequences of carrying large blood clones entailing other mutations is largely unknown. Our data demonstrates that CADASIL-related *NOTCH3*^*C455R*^ expression provides a selective advantage to *NOTCH3*^*C455R*^ HSPCs in vitro and in vivo. Remarkably, NOTCH3^C455R^*-*expressing hematopoietic cells provided a supportive environment for the expansion of *Dnmt3a*^*R878H*^ HSPCs in vivo (Supplementary Fig. 11). This suggests the intriguing possibility that CADASIL patients and asymptomatic carriers of CADASIL-related *NOTCH3* mutations may be particularly prone to *DNMT3A*^*R882H*^ mediated-CH than the general population.

Most CADASIL-*NOTCH3* variants swap cysteines for arginines leading to unpaired cysteines among the 34 EGFR [19], with more severe variants affecting domains 1–6 [19, 28]. The effect of cysteine-altering variants on NOTCH3 signaling activity is incompletely understood [40–45]. These mutations result in incomplete disulfide-bridge promoting the multimerization of NOTCH3 and emergence of granular osmiophilic deposits in CADASIL patients. CADASIL-NOTCH3-C455R variant has been reported as hypomorphic [20]. Supporting this, *Notch3*^−/^^−^ mice, which are highly susceptible to ischemic brain injury, can be rescued by the expression of *WT-NOTCH3* cDNA in VSMCs but not of *NOTCH3*^*C455R*^ cDNA [17, 20]. Yet, neomorphic properties cannot be excluded [20]. Remarkably, our data shows that NOTCH3-C455R expression confers a strong selective advantage to HSPCs both in vitro and in vivo (Figs. 1D, 2B, C, 3Aii, iii and Supplementary Fig. 3). This resulted in a dramatic accumulation of *NOTCH3*^*C455R*^ hematopoietic cells both in the BM and PB (Fig. 2B, C and Supplementary Fig. 3). *NOTCH3*^*C455R*^-GFP^+^ accumulation of PB cells did not exceed that of *NOTCH3*^*C455R*^ mutant HSPCs in the BM (Fig. 2B, C and Supplementary Fig. 3), indicating that the fitness advantage lays at the progenitor level. The expansion of *NOTCH3*^*C455R*^ mutant WBCs with time likely reflects the upstream accumulation of *NOTCH3*^*C455R*^ HSPCs that eventually contributed to differentiated blood cells [46]. Here, cellular expansion of HSPCs was likely due to faster cellular proliferation of *NOTCH3*^*C455R*^ HSCs as we observed in vitro (Fig. 3A). *NOTCH3*^*C455R*^-HSCs also displayed a transcriptional profile supportive for HSC expansion (Fig. 4D). Additionally, the accumulation of *NOTCH3*^*C455R*^-GFP^+^ MPPs, HPC-1 and HPC-2 cells does not surpass that of *NOTCH3*^*C455R*^-GFP^+^ HSCs (Fig. 2C and Supplementary Fig. 3), supporting that the CH fitness advantage lays at the HSCs. MPPs have been also shown recently to sustain lifelong hematopoiesis [47, 48] and could potentially contribute to the progressive accumulation of *NOTCH3*^*C455R*^ mutant hematopoietic cells over time. However, *NOTCH3*^*C455R*^-GFP^+^ MPPs, HPC-1, HPC-2 cells displayed lower levels of several cell cycle related genes (*Ccnb1, Ccnd2, Ccne1, Cdk1, Cdk2, Cdk7*) while we did not detect downregulation of these genes in *NOTCH3*^*C455R*^-GFP^+^ HSCs (Supplementary Fig. 9). In contrast, GSEA on bulk RNAseq data from *NOTCH3*^*C455R*^-HSCs, more sensitive than qRT-PCR, was able to detect enrichment of gene sets implicated in cell division in *NOTCH3*^*C455R*^-HSCs (Fig. 4D), further supporting their role as the main target cell for CH fitness advantage.

Particularly, *NOTCH3*^*C455R*^ mutant HSCs were enriched in multiple pathways that promote cell division including mTORC1-, TGF-β- TNFα-mediated NF-κB-signaling and MYC-target genes, which likely contribute to the observed upregulation of E2F-target genes and of cell cycle machinery components [49] (Fig. 4D). In particular, TNFα has been shown to stimulate HSC survival by triggering a NF-κB-dependent gene program which hinders necroptosis and biases HSCs for myeloid differentiation [50]. Likewise, mTORC1 activity drives the “proliferation, differentiation and long-lived maintenance” of HSC and HSPCs [51] and the MYC proto-oncogene is a critical regulator of cell proliferation and apoptosis and commonly deregulated in cancer [52]. Interestingly, HSC subtypes may respond differently to TGF-β, and while myeloid-biased HSCs proliferate under TGF-β, the cell growth of the lymphoid-biased HSC pool is constrained [53]. Additionally, the upregulation of hypoxia-related genes in *NOTCH3*^*C455R*^ mutant HSCs likely contributes to the efficient maintenance of *NOTCH3*^*C455R*^ HSCs as HSCs reside normally in a low-oxygen microenvironment in vivo [54]. Overall, *NOTCH3*^*C455R*^ mutant HSCs display a molecular profile that facilitates HSC expansion leading to CH and the development of a myeloid-bias HSC compartment, both characteristic of an aging hematopoietic system [3].

Paradoxically, *NOTCH*-activating mutations and constitutive activation of the NOTCH signaling pathway provide T-leukemic cells with a strong growth advantage [55]. Hence, the proliferative advantage of *NOTCH3*^*C455R*^ HSPCs could be due to a detrimental role of NOTCH3 signaling pathway in HSPC expansion which it is counteracted by hypomorphic NOTCH3-C455R. Alternatively and/or simultaneously, NOTCH3-C455R may provide a neomorphic function that promotes cell proliferation.

Co-expression of *NOTCH3*^*C455R*^ and *Dnmt3a*^*R878H*^ in HSPCs resulted in faster accumulation of double mutant hematopoietic cells in vitro (Fig. 1D) and in vivo in B-cells (Fig. 2B). Mutations in the R882 residue of *DNMT3A* are predominant in a broad range of hematological malignancies. For instance, ~25% of acute myeloid leukemia patients and ~15% of old individuals with CH carry *DNMT3A*^*R882*^ mutations (*DNMT3A*^*R882mut*^) [4, 6, 56]. *DNMT3A*^*R882mut*^ have been described both as loss-of-function (hypomorphic and acting as a dominant-negative) and gain-of-function depending on the biological context [56, 57]. Recent studies indicated a requirement of the dominant-negative effect of *DNMT3A*^*R882mut*^ to induce leukemogenesis [56]. *DNMT3A*^*R882mut*^ expression results in CpG hypomethylation mainly at hematopoietic enhancers [56, 58]. Consequently, DNMT3A was proposed as the “guardian of the epigenetic state preventing leukemic transformation” [58]. The molecular mechanisms through which DNMT3A prevents leukemogenesis are incompletely understood [58]. As we detected an additional growth advantage in *Dnmt3a*^*R878H*^ *NOTCH3*^*C455R*^ double mutant cells compared to single mutants (Figs. 1D, 2B, 3B), it is possible that both mutations operate over different molecular pathways in this cellular context or compensate specific molecular deficiencies. Interestingly, in a mouse model of chronic lymphocytic leukemia (CLL) where B1a cells are the leukemia cell of origin, *Dnmt3a* depletion resulted in the activation of NOTCH signaling and *Dnmt3a*-deficient CLL cells were sensitive to NOTCH signaling inhibition [59]. Yet, the functional interaction among DNMT3A and the NOTCH pathway may be cell type dependent and likely complicated by the presence of other NOTCH receptors. In our model, the co-expression of *NOTCH3*^*C455R*^ and *Dnmt3a*^*R878H*^ in HSPCs downregulated the expression of CIP/KIP cell cycle inhibitors (*p21*, *p27* and *p57*) present in single mutant *Dnmt3a*^*R878H*^ HSPCs (Supplementary Fig. 9A, B), which likely facilitates a more efficient cell cycle progression in double mutant HSPCs [60]. Interestingly, *NOTCH3*^*C455R*^ and *Dnmt3a*^*R878H*^ co-expression reverts the “*Dnmt3a*-mutant-signature” observed in single mutant *Dnmt3a*^*R878H*^ HSPCs (Supplementary Fig. 9). Thus, further investigations are needed to determine if *NOTCH3*^*C455R*^ facilitates or blocks *Dnmt3a*^*R878H*^ mediated leukemogenesis as *Dnmt3a*^*R878H*^ on its own is not able to drive transformation and requires the presence of mutations in other genes (e.g. *Npm1*) [34].

Most surprisingly, our data showed that single mutant *Dnmt3a*^*R878H*^ HSPCs displayed a strong selective advantage under the presence of *NOTCH3*^*C455R/C455R*^ hematopoietic cells in vivo (Fig. 3B). This phenotype was not observed in the presence of *NOTCH3*^*+/C455R*^ cells suggesting a NOTCH3-dose dependent effect (Fig. 3B). The molecular mechanism driving this non-cell autonomous selective advantage requires further investigation. Importantly, our findings suggest that CADASIL patients (at least those carrying *NOTCH3*^*C455R*^) may be particularly prone to *DNMT3A*^*R882H*^ clonal expansion due to this non-cell autonomous advantage provided by a *NOTCH3*^*C455R*^ hematopoietic cell milieu. More broadly, our data highlights the likely relevance of genetic variants on creating a microenvironment that supports CH development.

Importantly, as aforementioned the actual frequency of CADASIL-related *NOTCH3* variants in the general population is much higher than expected (~1 in 400) [19, 28–31]. Hence, considering this high prevalence of CADASIL-*NOTCH3* variants [19], and our data indicating that: (i) *NOTCH3*^*C455R*^ CADASIL mutations allow a selective advantage in HSPCs; and (ii) that the presence of *NOTCH3*^*C455R*^ hematopoietic cells provides a non-autonomous selective advantage to *DNMT3A*^*R882H*^ clones, it would very relevant to investigate the correlation of CADASIL-*NOTCH3* mutations in the general population and CH development. Particularly, there is no available data on the co-occurrence of *DNMT3A* and CADASIL-*NOTCH3* mutations.

In CADASIL, the degeneration of VSMCs and endothelial cells promotes vessel thrombosis, decreased blood flow and progressive disruption of the blood-brain barrier [61, 62]. Yet, the role of other cellular components is unknown. Recently NOTCH3 signaling has been shown as key in regulating inflammation by macrophage activation via NF‐κB [37]. Interestingly, our data indicated that *NOTCH3*^*C455R*^ CD11b^+^Gr1^−^ myeloid cells (comprising monocytes/macrophages) exhibited a perturbed inflammatory profile producing higher TNF-α levels (Fig. 4Fii). Exacerbated TNF-α production induces pleiotropic effects on various cell types contributing to inflammatory and autoimmune diseases, including atherosclerosis [38] in which macrophages play a key role by turning into foam cells that migrate into the subendothelial intimal space, which it is then invaded by VSMCS [63]. If *NOTCH3*^*C455R*^ macrophages and/or if brain resident *NOTCH3*^*C455R*^ microglial cells contribute to the damage of brain small vessels and facilitate CADASIL development requires further investigation. Additionally, the enhanced “TNF-α signaling via NF-κB” detected in *NOTCH3*^*C455R*^ HSCs (Fig. 4D) could suggest a positive feedback loop of TNF-α-producing *NOTCH3*^*C455R*^ myeloid cells on *NOTCH3*^*C455R*^ HSCs stimulating further the expansion of this HSC pool. Interestingly, we observed that in mice harboring a *NOTCH3*^*C455R*^ CADASIL-like hematopoietic system, blood vessels in the brain parenchyma were dilated (Fig. 4G), which could account for perturbations in blood flow regulation and the vessel integrity [39]. If this could promote an increased susceptibility to vascular-related conditions, such as edema or potential bleeding tendencies due to weakened vessel walls warrants further evaluation. Yet, we did not detect other CADASIL-related features.

So far, *NOTCH3* mutations have not been identified as CH-driver mutations in human patients [7]. Considering that *NOTCH3*^*C455R*^ provides a proliferative advantage to HSPCs in vivo it is possible that somatically acquired CADASIL-related *NOTCH*3 mutations in HSPCs could facilitate CH development. If CADASIL hematopoietic cells (e.g. macrophages) played any role in the development of vascular dementia, the presence of CADASIL-related CH could facilitate the development of vascular dementia in the general population.

Interestingly, ~50% of CADASIL patients die of pneumonia, excluding aspiration pneumonia [64], which suggests a compromised ability to respond to infections. Our transcriptomic analyses suggested perturbed transcriptional profiles in antigen processing and proteasome-related genes in *NOTCH3*^*C455R*^ T-cells and CD11b^+^ monocytes/macrophages, respectively (Fig. 4E, F). The proteasome plays a key role in innate and adaptive immune responses by degrading products that can be loaded onto major histocompatibility class I molecules for antigen presentation [65]. The potential relevance of these alterations needs further evaluation.

Overall, our data demonstrated that the expression of CADASIL-*NOTCH3*^*C455R*^ mutation provides a strong expansion advantage to HSPCs. Surprisingly, *NOTCH3*^*C455R*^ hematopoietic cells provided a “fertile soil” for the expansion of *Dnmt3a*^*R878H*^ mutant HSPCs in a non-cell autonomous fashion. Considering the high prevalence of CADASIL-related mutations in the general population, further studies are warranted to determine if CADASIL patients and those asymptomatic carriers of CADASIL-*NOTCH3* variants exhibit a higher risk of developing *DNMT3A*^*R882mut*^ clonal hematopoiesis.

## Supplementary information

Supplements Material and Methods

Supplemental Tables 1–3

Supplemental Table 4

## Data Availability

Additional methods and data can be found in the Supplementary section of this article. For original data, please contact the corresponding author m.ganuza@qmul.ac.uk.
