# Supplementary material for: A CADASIL NOTCH3 mutation leads to clonal hematopoiesis and expansion of Dnmt3a-R878H hematopoietic clones
Source: Leukemia. 2024 Nov 13;39(2):460–72. doi: 10.1038/s41375-024-02464-8 (PMC11794143; doi:10.1038/s41375-024-02464-8)
Supplement: Supplementary file 1 — Supplements Material and Methods [file 41375_2024_2464_MOESM1_ESM.pdf]

Supplemental Material and Methods

Supplemental Methods

Mice

Vav-iCre [B6.Cg-Commd10Tg(Vav1-icre)A2Kio/J, stock #:008610(1)], *NOTCH3*$^{fl-C455R-GFP}$ [129S-Gt(ROSA)26Sor $^{tm2(NOTCH3*C455R)Sat}$/Mmjax, MMRRC stock #033000-JAX(2)] and *Dnmt3a*$^{fl-R878H}$ [B6(Cg)-Dnmt3a^{tm1Trow}/J Strain #:032289(3)] were acquired from The Jackson Laboratory (Bar Harbor, Maine). *HSC-Scl-CRE-ERT* strain(4) was a kind gift of Dr. Joachim Göthert and the TELETHON KIDS INSTITUTE of Perth Children's Hospital, Nedlands, Western Australia (Telethon Kids). Mice of both sexes were used.

Tamoxifen delivery in vivo

To activate CRE *in vivo*, 8-10 weeks old mice were treated, respectively, for 1 or 5 consecutive days by oral gavage (20ga x 38mm plastic feeding tubes, Instech Laboratories) with 1mg (low induction) or 2mg (high induction) of tamoxifen (TAM, T5648, Sigma-Aldrich) suspended in 90% sunflower seed oil (Sigma-Aldrich) and 10% ethanol (Fisher BioReagents). Exact details for each tamoxifen regime are indicated within each figure and main text. Mice were treated with ~100ul or 200ul of a 10mg/ml TAM solution (*i.e.* ~40-80mg TAM/kg body weight), respectively.

Genotyping

PCRs were performed following manufacturer's instructions employing Go Taq G2 Flexi DNA Polymerase (Promega). PCR conditions: (95ºC, 2'); ((94ºC, 30''; 56ºC, 30''; 72ºC, 30''] × 35); (72ºC, 10'). Primers: Cre F1 (5'-

CTGTTACGTATAGCCGAAAT-3’), Cre R1(5’-CTACA CCAGAGACGGAAATC T-3’) were used to detect the *HSC-Scl-CRE-ERT* allele. CRE-positive PCR band: 203 bp. Detection of the GFP allele was used for $NOTCH3^{fl\text{-}C455R\text{-}GFP}$ allele genotyping: GFP Fw (5’-CAGATGAAGCAGCACGACTTCT-3’), GFP Rv (5’-AACTCCAGCAGGACCATGTGAT-3’). GFP PCR band: 400 bp. Primers VAViCre Fwd (5’-CCGAGGGGCCAAGTGAGAGG-3’) and VaviCre Rev (5’-GGAGGGCAGGCAGGTTTTGGTG-3’) were employed to detect the *VaviCre* allele. PCR band: ~300bp. To detect the $Dnmt3a^{fl\text{-}R878H}$ allele we employed the following primers obtained from Jackson laboratories: 41315-Dnmt3a Fw (5’-CTCCTTGGATTTGAGGAGGA-3’), 41316-Dnmt3a-wt-Rv (5’-TGCACATGAGAACTGGATGG-3’), oIMR7412-Dnmt3a-Mut-Rv (5’-ATTAAG GGCCAGCTCATTCC-3’). PCR bands: $Dnmt3a^{fl\text{-}R878H}$Mutant = 282 bp, *Dnmt3a* wild type=178bp.

PB Analysis

Mouse peripheral blood was collected in K2 EDTA-coated capillary tubes (microvette CB300, Sarstedt) from the lateral vein of the tail, lysed in red blood cell lysis buffer (Zen-Bio) and stained with the following antibodies used at 1:200 dilution: CD4-PECy7 (RM4-5) (Biolegend, San Diego, CA), CD8-PECy7 (53-6.7) (Biolegend), B220-PECy7 (RA3-6B2) (Biolegend), B220-PerCPCy5.5 (RA3-6B2) (Biolegend) Gr1-PerCPCy5.5 (RB6-8C5) (RA3-6B2) (Biolegend), CD11b-PerCPCy5.5 (M1/70) (RA3-6B2) (Biolegend). To assess cellular viability, 0.1µg/mL 4',6-diamidino-2-phenylindole (DAPI) staining was used. Analysis was performed on a LSR Fortessa I (BD Biosciences, San Diego, CA). Data was collected with BD FACSDiva Software

(version 8.0.1) (BD Biosciences, San Diego, CA) and analysed with FlowJo version 10.8.0 (BD Life Sciences).

Bone Marrow Analysis

Bone marrow was harvested from the femurs, tibias, pelvic bones and spines of mice by crushing with mortar and pestle. Cells were stained and analysed to identify: haematopoietic stem cells: HSCs (Lineage^{-}Sca-1^{+}c-Kit^{+} (LSK) CD150^{+}CD48^{-}); haematopoietic progenitor cell 1: HPC-1 (LSK CD150^{-}CD48^{+}); haematopoietic progenitor cell 2: HPC-2 (LSK CD150^{+}CD48^{+}); multipotent progenitors: MPP (LSK CD150^{-}CD48^{-}); multipotent progenitors-a: MPP-a (LSK CD135^{+}); common myeloid progenitors: CMP (Lineage^{-}c-Kit^{+}Sca-1^{-}CD32/16^{Low}CD34^{+}); common lymphoid progenitors: CLP (Lineage^{-}c-Kit^{Low}Sca-1^{Low}CD127^{+}); megakaryocyte-erythroid progenitors: MEP (Lineage^{-}c-Kit^{+}Sca-1^{-}CD32/16^{-}CD34^{-}); granulocyte-myeloid progenitors: GMP (Lineage^{-}c-Kit^{+}Sca-1^{-}CD32/16^{-}CD34^{-})]. The following antibodies were employed for the characterisation of HSCs, MPPs, HPC-1 and HPC-2: Lin-PerCP [Ter119 (TER119), CD4 (RM4-5), CD8 (53-6.7) , Gr1 (RB6-8C5), B220 (RA3-6B2), CD19 (6D5)] (all of them Biolegend), Sca-1-PE (E13-161.7) (Biolegend), c-Kit-APCe-780 (2B8) (Biolegend), CD48-A700 (HM48-1) (Biolegend), CD150-PECy7 (TC15-12F12.2) (Biolegend) and CD135-APC (A2F10) (Biolegend) (1:50). For the identification of CMP, CLP, MEP and GMP the following antibodies were used: Lin-PerCP [Ter119 (TER119), CD4 (RM4-5), CD8 (53-6.7) , Gr1 (RB6-8C5), B220 (RA3-6B2), CD19 (6D5)] (all of them Biolegend), Sca-1-PE (E13-161.7) (Biolegend), c-Kit-APCe-780 (2B8) (Biolegend); CD127-PECy7 (A7R34) (Biolegend)]; CD32/16-A700 (93) (Invitrogen eBioscience) andCD34-A647 (RAM34) (Biolegend) (1:50). All

antibodies were employed at 1:200 unless specified and 0.1µg/mL DAPI was used to exclude dead cells.

HSCs cultured *in vitro* (see below), were identified as LSK $CD150^+CD201/EPCR^+$ as recently described(5-8).

Cell cycle analysis

After staining for surface antigens, cells were fixed and permeabilised with Cytofix/Cytoperm Fixation / Permeabilization kit (BD Biosciences, San Diego, CA) and stained with Ki67-e660 (SolA15) (Invitrogen) and 0.02 µg/µL DAPI for cell cycle analysis. For HSCs, MPPs, HPC-1 and HPC-2 the following antibodies were used: Lin-PerCP [Ter119 (TER119), CD4 (RM4-5), CD8 (53-6.7) , Gr1 (RB6-8C5), B220 (RA3-6B2), CD19 (6D5)] (all of them Biolegend), Sca-1-PE (E13-161.7) (Biolegend), c-Kit-APCe-780 (2B8) (Biolegend), CD48-A700 (HM48-1) (Biolegend), CD150-PECy7 (TC15-12F12.2) (Biolegend), CD135-Biotin (A2F10) (Biolegend) (1:50) and streptavidin-BV605 (1:400). To study cell cycle in CMPs, CLPs, MEPs and GMPs the following antibodies were employed: Lin-PerCP [Ter119 (TER119), CD4 (RM4-5), CD8 (53-6.7) , Gr1 (RB6-8C5), B220 (RA3-6B2), CD19 (6D5)] (all of them Biolegend), Sca-1-Biotin (E13-161.7) (Biolegend), streptavidin-BV605 (Biolegend), c-Kit-APCe-780 (2B8) (Biolegend); CD127-PECy7 (A7R34) (Biolegend)]; CD32/16-A700 (93) (Invitrogen eBioscience) and CD34-PE (RAM34) (Biolegend) (1:50). For cell cycle *in vitro* studies in LSK cultures the staining was as follows: Lin-PerCP [Ter119 (TER119), CD4 (RM4-5), CD8 (53-6.7) , Gr1 (RB6-8C5), B220 (RA3-6B2), CD19 (6D5)] (all of them Biolegend), Sca-1-AF700 (E13-161.7) (Biolegend), c-Kit-APCe-780 (2B8) (Biolegend), CD150-PECy7 (TC15-12F12.2) (Biolegend), CD135-Biotin (A2F10) (Biolegend) (1:50), streptavidin-BV605 (1:400) and CD201 / EPCR-PE

(RMEPCR1560) (Stem cell technologies). All antibodies were employed at 1:200 unless specified. For cell cycle analysis, DAPI was measured on a linear scale.

Isolation of hematopoietic stem and progenitor cells (HSPCs)

Bone marrow cells were harvested from femurs, tibias, and pelvic bones of 8- to 10-week-old mice by bone crushing with a mortar and pestle. c-Kit$^+$ cells were enriched magnetically using anti-c-Kit microbeads (Miltenyi Biotech, San Diego, CA). Cells were then stained with fluorescently conjugated antibodies for lineage markers: B220 (RA3-6B2) (Biolegend), CD8 (53-6.7) (Biolegend), CD19 (6D5) (Biolegend), Gr-1 (RB6-8C5) (Biolegend), and Ter119 (TER-119) (Biolegend); Sca-1 (E13-161.7) and c-Kit (2B8). All antibodies were used at 1:200 dilution and 0.1µg/mL DAPI staining was used to exclude dead cells. Cells were sorted on a BD FACSAria II (BD Biosciences, San Diego, CA). Data was analysed as described above.

Statistics and Reproducibility:

Quantitative data are reported as means ± standard deviation from at least three different values. No randomization method was used. A Shapiro-Wilk normality test was first performed. Then, statistical significance between two different groups was determined using a two-tailored Student's t-test or Mann-Whitney's U test. To test for differences among the means of three or more groups a Turkey´s multiple comparison test was performed at a level of 0.05. p-values < 0.05 were considered statistically significant. Analyses were conducted in Graph Pad Prism 9. Sample size and number of experiment replicates are detailed in each Figure Legend. No statistical method was used to predetermine sample size. No data exclusion was carried out. Animals were allocated into experimental groups according to genotype. Although no specific methods were

used for blinding, blood and thymus samples were collected from mice by one individual and then analysed by flow cytometry by different individuals. Experiments were reliably reproduced. *In vivo* and *in vitro* results under low-induction TAM treatments were supported by independent high-induction. TAM treatments on separate cohorts of mice.

HSPC cell cultures

Mouse HSPCS (total LSK, HSCs or non-HSCs LSKs, as indicated) were sorted by FACS from bone marrow and cultured in serum-free expansion medium, as previously described(9, 10). Briefly, the medium contains Ham's F12 nutrient mix (Life technologies Invitrogen) supplemented with penicillin-streptomycin-glutamine (Gibco Invitrogen), insulin-transferrin-selenium-ethanolamine (Gibco Invitrogen), 10mM Hepes (Gibco Life Technologies Invitrogen), 1mg/mL poly-vinyl alcohol (Sigma), 100ng/mL recombinant mouse thrombopoietin (Preprotech) and 10ng/mL recombinant mouse stem cell factor BSA-free (Preprotech). Cells were cultured under these conditions for 21 days with passaging and addition of fresh culture medium every three days.

Quantitative real-time PCR analysis

Mouse HSPCs (HSC: Sca1+cKit+CD48-CD150+; MPP: Sca1+cKit+CD48-CD150-, HPC-1: Sca1+cKit+CD48+CD150- and HPC-2: Sca1+cKit+CD48+CD150+) and mouse blood cells (T cells: Gr1-CD11b-B220-CD4+CD8+; B cells: Gr1-CD11b-B220+CD4-CD8-; Gr1-CD11b+ cells and Gr1+CD11b- cells) were isolated by FACS from bone marrow and peripheral blood respectively. Total RNA from these cells was

isolated with RNeasy microKit (Qiagen) and 1μg RNA was reverse transcribed with High-capacity cDNA Reverse Transcription kit (Applied Biosystems) using random primers. Quantitative real-time polymerase chain reaction was performed in a C1000-Touch thermal cycler (BioRad) with SSO Advanced Universal SYBR Green Supermix (BioRad), as previously described(11). For the detection of each one of the corresponding mRNAs the following oligonucleotides were used: mouse-Notch3-Fw, 5’-CGTGTGGCCTCTTTCTACTGT-3’; mouse-Notch3-Rv, 5’-GCACCAATCGAGCACTCATC-3’; mouse-Ccnd2-Fw, 5’-GCAGAAGGACATCCAACCGTAC-3’; mouse-Ccnd2-Rv, 5’-ACTCCAGCCAAGAAACGGTCCA-3’; mouse-Ccnd3-Fw, 5’-GCGTGCAAAAGGAGATCAAGCC-3’; mouse-Ccnd3-Rv, 5’-CCAGGTAGTTCATAGCCAGAGG-3’; mouse-Ccna1-Fw, 5’-GCTACTGAGGATGGAGCATCTG-3’; mouse-Ccna1-Rv, 5’-CAGCTTCCAGAAGGCTCAGTTC-3’; mouse-Ccne1-Fw, 5’-AAGCCCTCTGACCATTGTGTCC-3’; mouse-Ccne1-Rv, 5’-CTAAGCAGCCAACATCCAGGAC-3’; mouse-Ccnb1-Fw, 5’-AGAGGTGGAACTTGCTGAGCCT-3’; mouse-Ccnb1-Rv, 5’-GCACATCCAGATGTTTCCATCGG-3’; mouse-Cdk6-Fw, 5’-ACCTCTGGAGTGTCGGTTGCAT-3’; mouse-Cdk6-Rv, 5’-TTCCTCTCCTGGGAGTCCAATG-3’; mouse-p21(Cdkn1a)-Fw, 5’-TCGCTGTCTTGCACTCTGGTGT-3’; mouse-p21(Cdkn1a)-Rv, 5’-CCAATCTGCGCTTGGAGTGATAG-3’; mouse-p57(Cdkn1c)-Fw, 5’-AGCTGAAGGACCAGCCTCTCTC-3’; mouse-p57(Cdkn1c)-Rv, 5’-ACGTCGTTCGACGCCTTGTTCT-3’; mouse-p27(Cdkn1b)-Fw, 5’-AGCAGTGTCCAGGGATGAGGAA-3’; mouse-p27(Cdkn1b)-Rv, 5’-

TTCTTGGGCGTCTGCTCCACAG-3’; mouse-Cdk1-Fw, 5’-CATGGACCTCAAGAAGTACCTGG-3’; mouse-Cdk1-Rv, 5’-CAAGTCTCTGTGAAGAACTCGCC-3’; mouse-Cdk2-Fw, 5’-TCATGGATGCCTCTGCTCTCAC-3’; mouse-Cdk2-Rv, 5’-TGAAGGACACGGTGAGAATGGC-3’; mouse-Cdc25a-Fw, 5’-CCTACTGATGGCAAGCGTGTCA-3’; mouse-Cdc25a-Rv, 5’-CTCATTGCCGAGCCTATCTCTC-3’; mouse-Cdc25b-Fw, 5’-CGCTCAAAGTCGCTGTGTCATG-3’; mouse-Cdc25b-Rv, 5’-GGTCTTGGTGTTTGCCATCCAC-3’; mouse-Cdk7-Fw, 5’-TGAGAATGGAGTTCTGAAACTGGC-3’; mouse-Cdk7-Rv, 5’-CCACACCATACATCCTAGCTCC-3’; mouse-Runx1-Fw, 5’-CACCGTCTTTACAAATCCGCCAC-3’; mouse-Runx1-Rv, 5’-CGCTCGGAAAAGGACAAACTCC-3’; mouse-Gata3-Fw, 5’-CCTCTGGAGGAGGAACGCTAAT-3’; mouse-Gata3-Rv, 5’-GTTTCGGGTCTGGATGCCTTCT-3’; mouse-Pbx1-Fw, 5’-CAACTCAGTGGAGCATTCCGAC-3’; mouse-Pbx1-Rv, 5’-GGCTTTGCTCTCGAAGGAGGTT-3’; mouse-Foxo1-Fw, 5’-CTACGAGTGGATGGTGAAGAGC-3’; mouse-Foxo1-Rv, 5’-CCAGTTCCTTCATTCTGCACTCG-3’; mouse-Nr4a2-Fw, 5’-CCGCCGAAATCGTTGTCAGTAC-3’; mouse-Nr4a2-Rv, 5’-TTCGGCTTCGAGGGTAAACGAC-3’; mouse-Vasn-Fw, 5’-CCAGCATCCATCTGCCTGAATG-3’; mouse-Vasn-Rv, 5’-CTTGCTCCACTGGACTCTCACA-3’; mouse-Prdm16-Fw, 5’-ATCCACAGCACGGTGAAGCCAT-3’; mouse-Prdm16-Rv, 5’-ACATCTGCCCACAGTCCTTGCA-3’; mouse-Flk2-Fw, 5’-

GTGCTGACGTTTGAAGACCTCC-3’; mouse-Flk2-Rv, 5’-GGTGACCAACACATTCCTGGCT-3’; mouse-Ikaros-Fw, 5’-CCACAACGAGATGGCAGAAGAC-3’; mouse-Ikaros-Rv, 5’-GGCATGTCTGACAGGCACTTGT-3’; mouse-Pu.1-Fw, 5’-GAGGTGTCTGATGGAGAAGCTG-3’; mouse-Pu.1-Rv, 5’-ACCCACCAGATGCTGTCCTTCA-3’; and mouse-Mef2c-Fw, 5’-GTGGTTTCCGTAGCAACTCCTAC-3’; mouse-Mef2c-Rv, 5’-GGCAGTGTTGAAGCCAGACAGA-3’. The oligonucleotides used for normalisation were Tbp-Fw-1 (5’-CTACCGTGAATCTTGGCTGTAAAC-3’), Tbp-Rv-1 (5’-AATCAACGCAGTTGTCCGTGGC-3’). Relative expression levels were analysed by the ΔΔCt mehod. Results are expressed as fold changes taking the HSC sample as a reference value of 1 in Supplemental Figure 1A or the HSC, MPP, HPC1 and HPC2 control samples in Supplemental Figure 9.

Transcriptional profiling (RNAseq) and mRNA expression analysis

For the construction of the non-directional library, messenger RNA was purified from total RNA using poly-T oligo-attached magnetic beads. After fragmentation, the first strand cDNA was synthesized using random hexamer primers, the second strand cDNA synthesis was carried out with dTTP followed by end repair, A-tailing, adapter ligation, size selection, amplification and purification. The library preparations were sequenced on an Illumina NovaSeq6000 platform with an average paired-end read length of 150 bp. mRNA expression and gene set enrichment analysis were performed using the Novogene developed platform NovoMagic (https://www.novogene.com/eu-en/novomagic-online-rna-seq-bioinformatics-analysis-t; NovoMagic—Novogene’s Online RNA-seq Bioinformatics Analysis Tool. https://www.novogene.com/us-

en/resources/onlineevent/introducing-novomagic-novogenes-online-rna-seq-bioinformatics-analysis-tool/#). Raw reads were mapped to the mouse genome (mmu,GRCm38). To determine differentially expressed genes, p-adjusted values < 0.05 were considered statistically significant and log2fold changes <-1 and >1 were considered. For gene set enrichment analysis (GSEA) FDR-q-values <0.25 were considered statistically significant. The complete RNAseq data is deposited as Sequence Read Archive (SRA) in the NCBI servers (BioProject ID: PRJNA1162818).

Colony genotyping of Dnmt3a^{R878H} mutant colonies.

HSPCs were sorted by FACS as indicated above and plated in M3434 methylcellulose (STEMCELL Technologies, Vancouver, Canada) to allow clonal expansion. Individual colonies were picked 10 days after plating and DNA isolated by digestion in 30ul of 50mM NaOH/0.2mM EDTA buffer for 1 hour at 98°C. After cooled down, 2ul were employed for genotyping. Two PCRs were conducted as previously described(3): PCR (F1+R1) enabling detection of the Wild-type allele (WT, PCR-band~325bp) and recombined mutant allele (*Dnmt3a-R878H,* PCR-band ~350bp). PCR (F2+R1) to detect the inducible floxed allele (fl-*Dnmt3a-R878H.* PCR-band *~225bp)*). PCR reactions were run in 2% Agarose for PCR (F2+R1) and 4% agarose gel for PCR (F1+R1). Dnmt3aR878H-F1 (5'-CCACTAGAACCCTCAGCACA-3'), Dnmt3aR878H-F2 (5'-AGTAAGTCTGCAGGTCGAGG-3'), Dnmt3aR878H-R1 (5'-CCCCAGACCTTTGAAATGCC-3').

Histopathology

Tissues fixed in 10%-buffered formalin (Sigma) were embedded in paraffin. Haematoxylin and eosin (bluing reagent) (#760-2021 and #760-2037, respectively;

Roche Diagnosis, Ltd) staining was carried out automatedly in a Leica Autostainer XL and mounted in a Leica CV5030 Cover-slipper (Leica, Wetzlar, Germany).

Analyses of brain blood vessels

Brain blood vessels were analyzed from similar areas in the brain parenchyma and typical cross-sections were captured. Feret's diameters were evaluated by ImageJ to determine vessels sizes. Particularly, minimum Feret's diameters, which is the smallest diameter of the vessel perpendicular to the maximum diameter, were compared among mouse cohorts.

Supplemental Tables

Supplemental Table 1 contains the sequences of the primers used for genotyping the mice. Supplemental Table2 includes the sequences of the primers used for quantitative Real Time PCR. Supplemental Table 3 presents the sequences of the primers used to genotype the DNMT3a colonies. Supplemental Table 4 contains all source data.

Supplemental Figure Legends

Supplemental Figure 1. *Notch3* mRNA expression is upregulated in HSCs and MPPs.

qRT-PCR from mRNAs isolated from mouse HSC, MPP, HPC-1 and HPC-2 sorted from WBM and from PB myeloid cells ($CD11b^{+}Gr1^{-}$, $CD11b^{-}Gr1^{+}$), B- and T-lymphoid cells. Representative sorting strategy in PB myeloid, B-cells, T-cells **(Ai)** and BM HSC, MPP, HPC-1, HPC-2 **(Aiii)**. Post-sort purity checks are provided **(Aii & Aiv)**. **Av.** qRT-PCR showed that *Notch3* mRNA is upregulated in HSCs and MPP cells. Standard deviations show variation among technical replicates (n=3)**.** Parametric

unpaired two-tailed t-test. **B.** *NOTCH3* mRNA expression data in the human hematopoietic system was retrieved from BloodSpot 3.0 (http://www.fobinf.com/)(12) collated from publicly available human datasets **(**GSE24759(13)). *NOTCH3* mRNA levels were higher in human HSPC (CD133^{+} CD34^{dim} and CD38^{-} CD34^{+}) cells. HSPC CD133^{+} CD34^{dim} n=10; HSPC CD38^{-} CD34^{+}, GMP, CMP, Early B cellm B cells, CD8+Effec.Mem.RA, NKT, CFU-Mono, Gran.(Neutro Metamyelo) and Granulo (Neutro) n=4; MEP, NK CD56^{-}CD16^{+} n=9; Pro B-cell, Naive B-cells, B-cells, NK CD56^{-}CD16^{-}, Monocyte, Eosinophill, CFU-Granulo, DC, CFU-Megakaryo n=5; Naive T-cells, Megakaryocytes n=7; CD8^{+}Effec.Mem., Eryth n=13; CD4^{+}Effec.Mem. and Eryth.CD34^{+}CD71^{+}GlyA^{-} n=14. One-way ANOVA, Tukey's multiple comparisons test. Means and standard deviations are indicated. **Av.** p-values relate to differences with HSC (upper stars) and MPP (lower stars) **(Av)** and with HSPC (CD133^{+} CD34^{dim}) **(B)**. **** $p < 0.001$, *** $p < 0.001$, ** $p < 0.01$, *$p < 0.05$. Source data in Supplemental Table 4.

Supplemental Figure 2. ***Gating strategies on peripheral blood and bone marrow hematopoietic compartments. Genotyping strategy to identify Dnmt3aR878H mutant colonies.***

A. Representative gating strategy in PB myeloid, B-cells, T-cells and in the bone marrow for HSPCs employed for flow cytometry analyses. **B.** Representative gating strategy to define cell cycle phases and to detect apoptotic cells. **C.** Genotyping strategy to identify mutational status for the *Dnmt3a*^{R878H} knock-in allele. Two PCRs were conducted as previously described(3). Wild-type allele (WT), inducible floxed allele (fl-*Dnmt3a-R878H)* and recombined mutant allele (*Dnmt3a-R878H*) are shown. Primers used for each PCRs are indicated (F2, F1 and R1). Representative agarose gels are

shown [2% agarose for PCR (primers: F2+R1) and 4% agarose gel for PCR (primers: F1+R1)].

Supplemental Figure 3. NOTCH3-C455R expression confers a fitness advantage to HSPCs *in vivo*.

Related to Figure 2C. Changes in the % of NOTCH3-C455R-GFP^{+} cells are shown for HSPCs from one week post-TAM to one year post-TAM in TAM-treated *HSC-Scl-CRE-ERT*$^{+/CRE}$*;NOTCH3*$^{C455R/+}$ and *HSC-Scl-CRE-ERT*$^{+/CRE}$*;NOTCH3*$^{C455R/C455R}$ mice in a *Dnmt3a*$^{+/R878H}$ or *Dnmt3a*$^{+/+}$ background. *NOTCH3*^{C455R}-GFP^{+} HSPCs accumulate over time. Means and standard deviations are indicated. **** p < 0.001, *** p < 0.001, ** p < 0.01, *p < 0.05. Parametric unpaired two-tailed t-test and two-way ANOVA, Tukey's multiple comparisons test. **Number of replicates:** same as in Figure 2C. Source data in Supplemental Table 4.

Supplemental Figure 4. Analysis on cellular and biochemical blood parameters in mice harboring *NOTCH3*^{C455R} and *Dnmt3a*^{R878H} alleles.

Blood tests on one year old TAM-treated *HSC-Scl-CRE-ERT*$^{+/CRE}$*;NOTCH3*$^{C455R/+}$ and *HSC-Scl-CRE-ERT*$^{+/CRE}$*;NOTCH3*$^{C455R/C455R}$ mice in *Dnmt3a*$^{+/R878H}$ and *Dnmt3a*$^{+/+}$ backgrounds following low-induction **(A)** and high-induction **(B)**. Related to Figure 2B-C. Means and standard deviations are indicated. **** p < 0.001, *** p < 0.001, ** p < 0.01, *p < 0.05. **Number of replicates-A**. Untreated–*HSC-Scl-CRE-ERT*$^{+/CRE}$*;NOTCH3*$^{+/C455R}$*;Dnmt3a*$^{+/+}$ n=3; TAM–*HSC-Scl-CRE-ERT*$^{+/+}$*;NOTCH3*$^{C455R/C455R}$*;Dnmt3a*$^{+/R878H}$ n=5; TAM–*HSC-Scl-CRE-ERT*$^{+/CRE}$*;NOTCH3*$^{+/+}$*;Dnmt3a*$^{+/+}$ n=3; TAM–*HSC-Scl-CRE-ERT*$^{+/CRE}$*;NOTCH3*$^{+/+}$*;Dnmt3a*$^{+/R878H}$ n=2; TAM–*HSC-Scl-CRE-*

ERT$^{+/CRE}$*;NOTCH3*$^{+/C455R}$*;Dnmt3a*$^{+/+}$ n=4; TAM–*HSC-Scl-CRE-ERT*$^{+/CRE}$*;NOTCH3*$^{+/C455R}$*;Dnmt3a*$^{+/R878H}$ n=3; TAM–*HSC-Scl-CRE-ERT*$^{+/CRE}$*;NOTCH3*$^{C455R/C455R}$*;Dnmt3a*$^{+/+}$ n=3; TAM–*HSC-Scl-CRE-ERT*$^{+/CRE}$*;NOTCH3*$^{C455R/C455R}$*;Dnmt3a*$^{+/R878H}$ n=3. **Number of replicates-B**. Untreated–*HSC-Scl-CRE-ERT*$^{+/CRE}$*;NOTCH3*$^{+/C455R}$*;Dnmt3a*$^{+/+}$ n=3; TAM–*HSC-Scl-CRE-ERT*$^{+/+}$*;NOTCH3*$^{C455R/C455R}$*;Dnmt3a*$^{+/R878H}$ n=5; TAM–*HSC-Scl-CRE-ERT*$^{+/CRE}$*;NOTCH3*$^{+/+}$*;Dnmt3a*$^{+/+}$ n=7; TAM–*HSC-Scl-CRE-ERT*$^{+/CRE}$*;NOTCH3*$^{+/+}$*;Dnmt3a*$^{+/R878H}$ n=1; TAM–*HSC-Scl-CRE-ERT*$^{+/CRE}$*;NOTCH3*$^{+/C455R}$*;Dnmt3a*$^{+/+}$ n=6; TAM–*HSC-Scl-CRE-ERT*$^{+/CRE}$*;NOTCH3*$^{+/C455R}$*;Dnmt3a*$^{+/R878H}$ n=4; TAM–*HSC-Scl-CRE-ERT*$^{+/CRE}$*;NOTCH3*$^{C455R/C455R}$*;Dnmt3a*$^{+/+}$ n=4; TAM–*HSC-Scl-CRE-ERT*$^{+/CRE}$*;NOTCH3*$^{C455R/C455R}$*;Dnmt3a*$^{+/R878H}$ n=5. Parametric unpaired two-tailed t-test. Source data in Supplemental Table 4.

Supplemental Figure 5. *In vivo* analysis of cell cycle profiles and HSPC populations in *NOTCH3*^{C455R} mice.

Related to Figure 2D. **A.** Experimental approach. *HSC-Scl-CRE-ERT*$^{+/CRE}$*;NOTCH3*$^{C455R/+}$ (shorthanded as *+/CRE;NOTCH3*$^{C455R/+}$) and *+/CRE;NOTCH3*$^{C455R/C455R}$ mice in a *Dnmt3a*$^{+/R878H}$ and *Dnmt3a*$^{+/+}$ background were TAM-treated to induce NOTCH3-C455R and DNMT3A-R878H expression in HSPCs *in vivo*. Number of replicates: same as in Figure 2D. **B-C.** Analysis of the cellular composition of GFP^{+} *NOTCH3*^{C455R} and GFP^{-} control HSPCs in TAM-treated *+/CRE;NOTCH3*$^{C455R/+}$ and *+/CRE;NOTCH3*$^{C455R/C455R}$ mice in *Dnmt3a*$^{+/R878H}$ and *Dnmt3a*$^{+/+}$ backgrounds one year post-TAM. Results are shown for high **(B)** and low **(C)** tamoxifen induction. HSPCs were analyzed for the presence of HSC, MPP, HPC-

1&2, CMP, GMP, MEP and CLP. Parametric unpaired two-tailed t-test. Means and standard deviations are indicated. ** $p < 0.01$, *$p < 0.05$. Source data in Supplemental Table 4. **Number of replicates-C:** TAM–*HSC-Scl-CRE-ERT*$^{+/CRE}$;*NOTCH3*$^{+/C455R}$;*Dnmt3a*$^{+/+}$ n=6; TAM–*HSC-Scl-CRE-ERT*$^{+/CRE}$;*NOTCH3*$^{C455R/C455R}$;*Dnmt3a*$^{+/+}$ n=3; TAM–*HSC-Scl-CRE-ERT*$^{+/CRE}$;*NOTCH3*$^{+/C455R}$;*Dnmt3a*$^{+/R878H}$ n=3; TAM–*HSC-Scl-CRE-ERT*$^{+/CRE}$;*NOTCH3*$^{C455R/C455R}$;*Dnmt3a*$^{+/R878H}$ n=5.

Supplemental Figure 6. Analysis of cell cycle profiles and HSPC populations in *NOTCH3*^{C455R} mice *in vivo*.

Related to Figure 3A. BM-HSPCs from TAM-treated *HSC-Scl-CRE-ERT*$^{+/CRE}$;*NOTCH3*$^{C455R/C455R}$ mice in a *Dnmt3a*$^{+/R878H}$ and *Dnmt3a*$^{+/+}$ background were analyzed by flow cytometry for cell cycle distribution via Ki67/DAPI staining and for apoptosis levels via AnnexinV/DAPI staining one week post-TAM treatment. **A.** Experimental schematic. **B.** Cell cycle distribution (G0, G1, S, G2/M phases) among HSPC populations (*i.e.* HSC, MPP, HPC-1&2, CMP, GMP, MEP and CLP). **C.** Frequency of apoptotic cells. Means and standard deviations are indicated. No relevant significant statistical differences were found. Parametric unpaired two-tailed t-test. Source data in Supplemental Table 4. **Number of replicates:** TAM–*HSC-Scl-CRE-ERT*$^{+/CRE}$;*NOTCH3*$^{C455R/C455R}$;*Dnmt3a*$^{+/+}$ n=5 for total, LSK, HSC, HPC1, HPC2, MPP and MPPa; for CLP, CMP, GMP and MEP n=4. TAM–*HSC-Scl-CRE-ERT*$^{+/CRE}$;*NOTCH3*$^{C455R/C455R}$;*Dnmt3a*$^{+/R878H}$ n=6 for total, LSK, HSC, HPC1, HPC2, MPP and MPPa; for CLP, CMP, GMP and MEP n=5.

Supplemental Figure 7. Cell cycle and phenotypic analysis of mono-cultures and co-cultures of *NOTCH3*^{C455R} mutant and control HSPCs.

Related to Figure 3A. Analysis of the cell cycle profiles **(A)**, apoptosis **(B)** and cellular composition **(C)** of mono-cultures and co-cultures of GFP^{+} *NOTCH3*^{C455R} and GFP^{-} control LSK cells isolated from TAM-treated (three doses of 2mg of TAM during three consecutive days) *HSC-Scl-CRE-ERT*$^{(+/CRE)}$*;NOTCH3*$^{C455R/+}$ and *HSC-Scl-CRE-ERT*$^{(+/CRE)}$*;NOTCH3*$^{C455R/C455R}$ mice in *Dnmt3a*$^{+/R878H}$ and *Dnmt3a*$^{+/+}$ backgrounds. Cells were sorted one-week post-treatment, cultured for 21 days and then analyzed. Sorted *NOTCH3*^{C455R}-GFP^{+} and GFP^{-} LSK cells were cultured either as mono-cultures or co-cultured. **A.** Cell cycle profiles (% of G0, G1, S, G2/M phases) analyzed by flow cytometry, following Ki67/DAPI staining, are depicted for LSK cells 21 days post-culture from both monocultures and co-cultures. **B.** Apoptotic levels were analyzed by AnnexinV/DAPI staining among LSK HSPCs. **C.** HSPCs were analyzed for the presence of HSC, MPP, HPC-1&2, CMP, GMP, MEP and CLP. **A-C.** Means and standard deviations are indicated. Parametric unpaired two-tailed t-test. No relevant significant statistical differences were found. Source data in Supplemental Table 4. **Number of replicates-A:** TAM–*HSC-Scl-CRE-ERT*$^{+/CRE}$*;NOTCH3*$^{+/C455R}$*;Dnmt3a*$^{+/+}$ n=3 from three independent experiments; TAM–*HSC-Scl-CRE-ERT*$^{+/CRE}$*;NOTCH3*$^{C455R/C455R}$*;Dnmt3a*$^{+/+}$ n=3 from 3 different experiments and n=3 for co-cultures from two independent experiments. TAM–*HSC-Scl-CRE-ERT*$^{+/CRE}$*;NOTCH3*$^{+/C455R}$*;Dnmt3a*$^{+/R878H}$ n=6 for GFP^{-} and n=7 for GFP^{+} from 5 independent experiments for monocultures and n= 4 for co-cultures from 4 independent experiments. TAM–*HSC-Scl-CRE-ERT*$^{+/CRE}$*;NOTCH3*$^{C455R/C455R}$*;Dnmt3a*$^{+/R878H}$ n=7 for monocultures from 4 independent experiments and n=5 for co-cultures from 3

independent experiments. **Number of replicates-B-C:** TAM–*HSC-Scl-CRE-ERT*$^{+/CRE}$*;NOTCH3*$^{+/C455R}$*;Dnmt3a*$^{+/+}$ for monocultures n=3 for GFP$^{–}$ and n=4 for GFP^{+} from three independent experiments. For co-cultures n=5 for both GFP$^{–}$ and GFP^{+} from 3 independent experiments. TAM–*HSC-Scl-CRE-ERT*$^{+/CRE}$*;NOTCH3*$^{+/C455R}$*;Dnmt3a*$^{+/R878H}$ n=5 for both GFP$^{–}$ and GFP^{+} monocultures from 4 independent experiments. For GFP$^{–}$ co-cultures, n=7 and for GFP^{+} co-cultures n=5. TAM–*HSC-Scl-CRE-ERT*$^{+/CRE}$*;NOTCH3*$^{C455R/C455R}$*;Dnmt3a*$^{+/+}$ for GFP$^{–}$ monocultures n=9 from 7 independent experiments and for GFP^{+} monocultures n= 12 from 10 independent experiments. For both GFP$^{–}$ and GFP^{+} cocultures n=7 from 4 independent experiments. TAM–*HSC-Scl-CRE-ERT*$^{+/CRE}$*;NOTCH3*$^{C455R/C455R}$*;Dnmt3a*$^{+/R878H}$ n=7 for both GFP$^{–}$ and GFP+ monocultures from 5 independent experiments. For GFP$^{–}$ and GFP^{+} co-cultures n=5 from 4 independent experiments.

Supplemental Figure 8. Blood tests in mice with widespread expression of *NOTCH3*^{C455R} in their hematopoietic system.

Blood tests in one year old *Vav1CRE*$^{+/CRE}$*;NOTCH3*$^{C455R/+}$ and *Vav1CRE*$^{+/CRE}$*;NOTCH3*$^{C455R/C455R}$. Related to Figure 4B-C. Means and standard deviations are indicated. Parametric unpaired two-tailed t-test. No significant statistical differences were found. *Vav1CRE*$^{+/+}$*;NOTCH3*$^{+/C455R}$ n=9; *Vav1CRE*$^{+/CRE}$*;NOTCH3*$^{+/C455R}$ n=10; *Vav1CRE*$^{+/CRE}$*;NOTCH3*$^{C455R/C455R}$ n=5 for WBC, RBC, HGB, HCT, MCV, MCH, PLT, RDWCV, RDWSD, PCT, MPV and MCHC. For PDW *Vav1CRE*$^{+/+}$*;NOTCH3*$^{+/C455R}$ n=9; *Vav1CRE*$^{+/CRE}$*;NOTCH3*$^{+/C455R}$ n=7; *Vav1CRE*$^{+/CRE}$*;NOTCH3*$^{C455R/C455R}$ n=5. Source data in Supplemental Table 4.

Supplemental Figure 9. Molecular interaction among DNMT3A-R878H and NOTCH3-C455R.

qRT-PCR from mRNAs isolated from HSC **(A)**, MPP **(B)**, HPC-1 **(C)** and HPC-2 **(D)** sorted from the WBM of TAM-treated *HSC-Scl-CRE-ERT*$^{+/CRE}$*;NOTCH3*$^{C455R/C455R}$ mice in *Dnmt3a*$^{+/R878H}$ and *Dnmt3a*$^{+/+}$ backgrounds and from control TAM-treated *HSC-Scl-CRE-ERT*$^{+/CRE}$*;NOTCH3*$^{+/+}$, *HSC-Scl-CRE-ERT*$^{+/+}$*;NOTCH3*$^{C455R/C455R}$ and *HSC-Scl-CRE-ERT*$^{+/+}$*;NOTCH3*$^{C455R/C455R}$*;Dnmt3a*$^{+/R878H}$ mice and untreated *NOTCH3*$^{C455R/C455R}$ mice. The transcript levels of cell cycle related genes **(Ai, Bi, Ci, Di)** and Dnmt3a related genes previously described to be perturbed in a *Dnmt3a*$^{-/-}$ background(14) as a "Dnmt3a-fingerprint" were analyzed **(Aii, Bii, Cii, Dii)**, including multipotency genes, HSC fingerprint and genes upregulated during differentiation. **A-D)** Means and standard deviations are indicated. Standard deviations show variation among biological replicates (n=3). Each biological replicate was pooled from at least 2 mice of the same genotype to obtain sufficient mRNA**.** Parametric unpaired two-tailed t-test. **** $p < 0.001$, *** $p < 0.001$, ** $p < 0.01$, *$p < 0.05$. Source data in Supplemental Table 4.

Supplemental Figure 10. Analysis of the blood vessels in the brain parenchyma of mice harboring a CADASIL-like hematopoietic system.

Related to Figure 4G. Hematoxylin & Eosin staining of the blood vessels analyzed and measured in the brain parenchyma of *Vav1CRE*$^{+/CRE}$*;NOTCH3*$^{C455R/C455R}$ (n=5) and *NOTCH3*$^{C455R/C455R}$ control mice (n=8). All the typical cross-sections analyzed, captured and measured in Figure 4G are shown.

Supplemental Figure 11. Summary of *NOTCH3*^{C455R} effects on the fitness of HSPCs.

NOTCH3^{C455R} expression confers a fitness advantage to HSPCs due to the faster proliferation of *NOTCH3*^{C455R} HSPCs. *Dnmt3a*^{R878H} and *NOTCH3*^{C455R} co-expression enhances the fitness of *NOTCH3*^{C455R} HSPCs. *NOTCH3*^{C455R} HSPCs confer a non-cell autonomous advantage to *DNMT3A*^{R878H} mutant HSPCs resulting in their cellular expansion.

References

1. de Boer J, Williams A, Skavdis G, Harker N, Coles M, Tolaini M, et al. Transgenic mice with hematopoietic and lymphoid specific expression of Cre. Eur J Immunol. 2003;33(2):314-25.
2. Arboleda-Velasquez JF, Manent J, Lee JH, Tikka S, Ospina C, Vanderburg CR, et al. Hypomorphic Notch 3 alleles link Notch signaling to ischemic cerebral small-vessel disease. Proc Natl Acad Sci U S A. 2011;108(21):E128-35.
3. Loberg MA, Bell RK, Goodwin LO, Eudy E, Miles LA, SanMiguel JM, et al. Sequentially inducible mouse models reveal that Npm1 mutation causes malignant transformation of Dnmt3a-mutant clonal hematopoiesis. Leukemia. 2019;33(7):1635-49.
4. Gothert JR, Gustin SE, Hall MA, Green AR, Gottgens B, Izon DJ, Begley CG. In vivo fate-tracing studies using the Scl stem cell enhancer: embryonic hematopoietic stem cells significantly contribute to adult hematopoiesis. Blood. 2005;105(7):2724-32.
5. Papa L, Djedaini M, Martin TC, Zangui M, Beaumont KG, Sebra R, et al. Limited Mitochondrial Activity Coupled With Strong Expression of CD34, CD90 and EPCR Determines the Functional Fitness of ex vivo Expanded Human Hematopoietic Stem Cells. Front Cell Dev Biol. 2020;8:592348.
6. Kohlscheen S, Schenk F, Rommel MGE, Cullmann K, Modlich U. Endothelial protein C receptor supports hematopoietic stem cell engraftment and expansion in Mpl-deficient mice. Blood. 2019;133(13):1465-78.
7. Fares I, Chagraoui J, Lehnertz B, MacRae T, Mayotte N, Tomellini E, et al. EPCR expression marks UM171-expanded CD34(+) cord blood stem cells. Blood. 2017;129(25):3344-51.
8. Balazs AB, Fabian AJ, Esmon CT, Mulligan RC. Endothelial protein C receptor (CD201) explicitly identifies hematopoietic stem cells in murine bone marrow. Blood. 2006;107(6):2317-21.
9. Wilkinson AC, Ishida R, Kikuchi M, Sudo K, Morita M, Crisostomo RV, et al. Long-term ex vivo haematopoietic-stem-cell expansion allows nonconditioned transplantation. Nature. 2019;571(7763):117-21.
10. Wilkinson AC, Ishida R, Nakauchi H, Yamazaki S. Long-term ex vivo expansion of mouse hematopoietic stem cells. Nat Protoc. 2020;15(2):628-48.
11. Holmfeldt P, Pardieck J, Saulsberry AC, Nandakumar SK, Finkelstein D, Gray JT, et al. Nfix is a novel regulator of murine hematopoietic stem and progenitor cell survival. Blood. 2013;122(17):2987-96.
12. Gislason MH, Demircan GS, Prachar M, Furtwangler B, Schwaller J, Schoof EM, et al. BloodSpot 3.0: a database of gene and protein expression data in normal and malignant haematopoiesis. Nucleic Acids Res. 2024;52(D1):D1138-D42.
13. Novershtern N, Subramanian A, Lawton LN, Mak RH, Haining WN, McConkey ME, et al. Densely interconnected transcriptional circuits control cell states in human hematopoiesis. Cell. 2011;144(2):296-309.
14. Challen GA, Sun D, Jeong M, Luo M, Jelinek J, Berg JS, et al. Dnmt3a is essential for hematopoietic stem cell differentiation. Nat Genet. 2011;44(1):23-31.

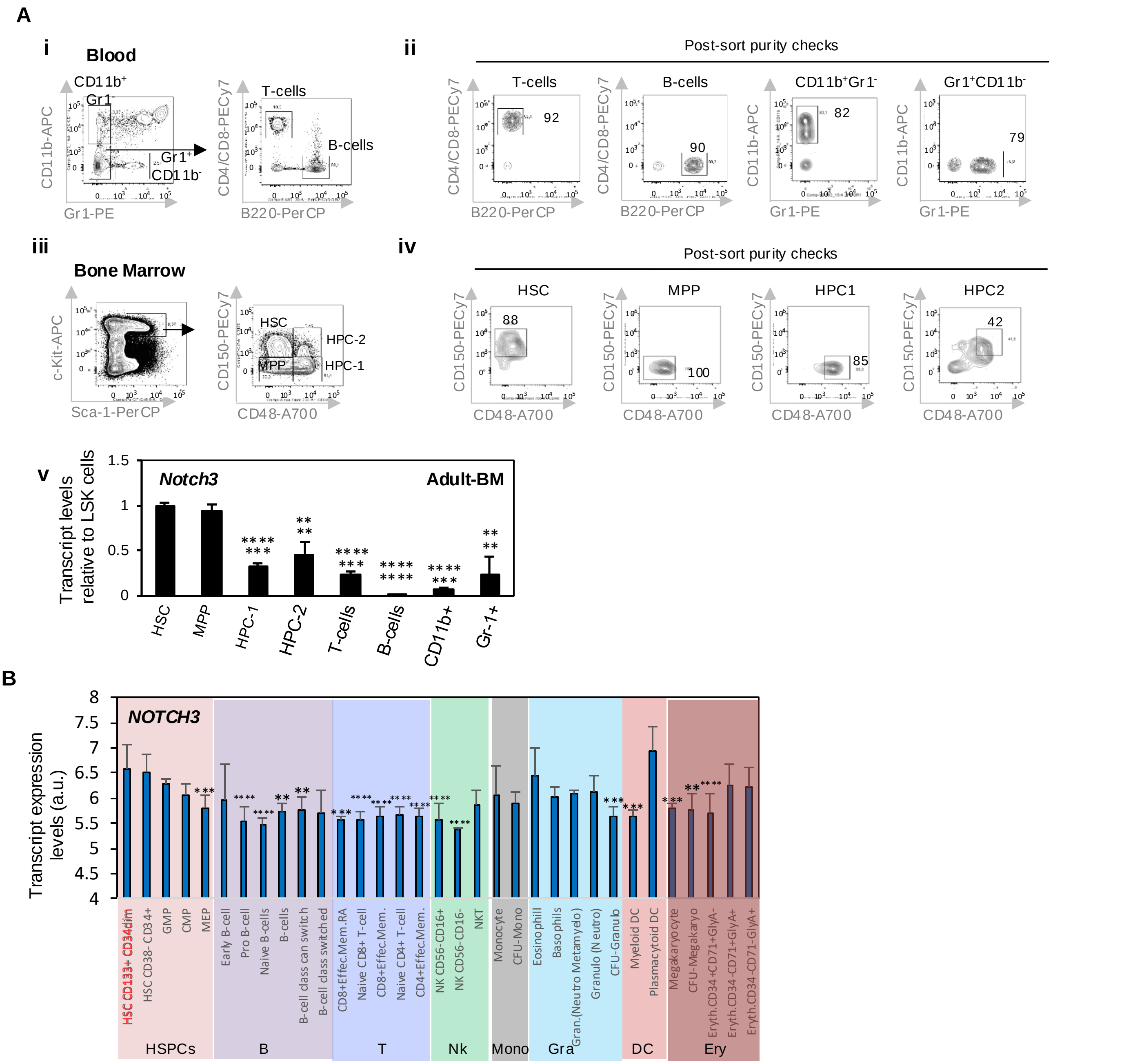

Supplemental Figure 1

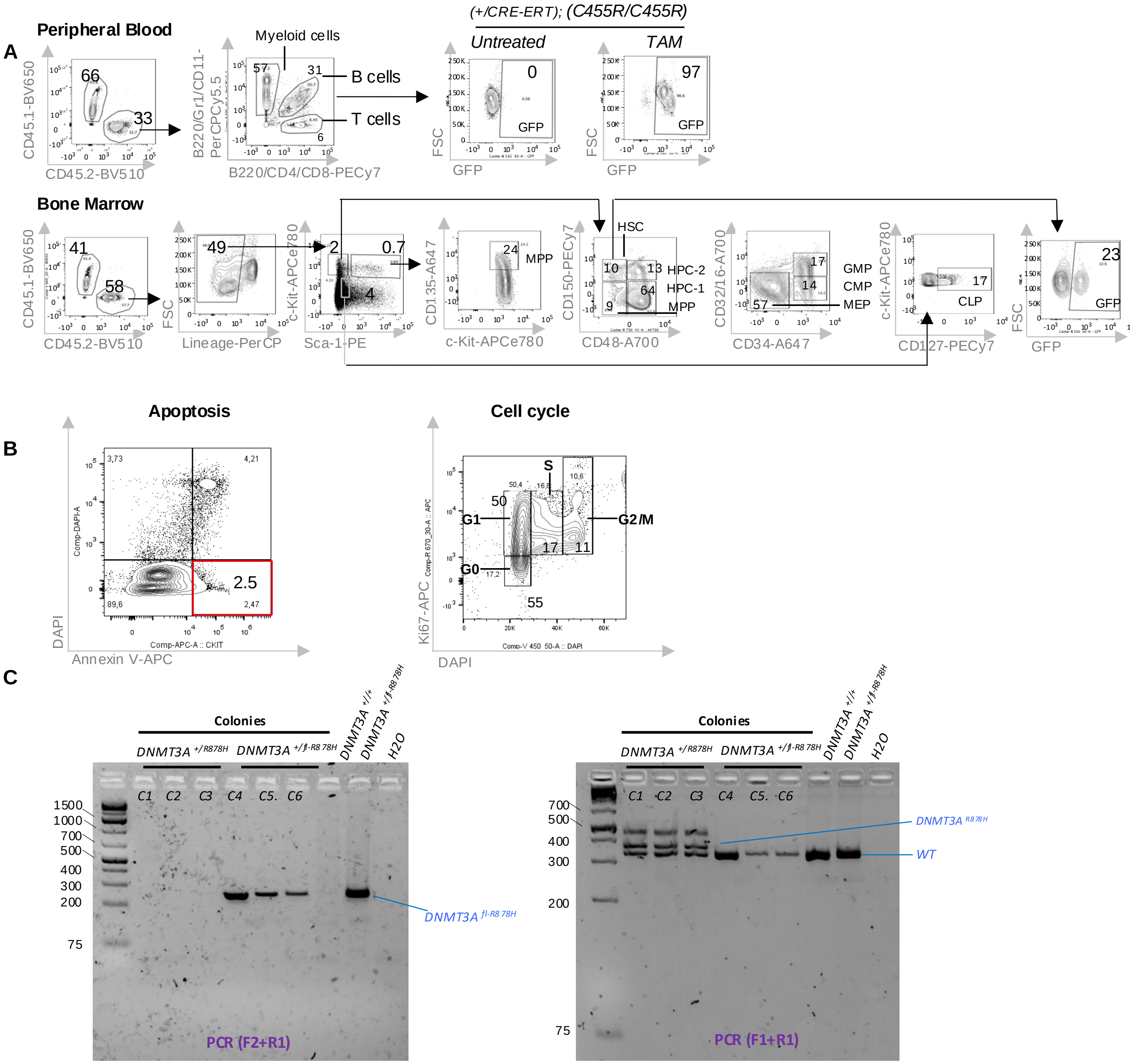

Supplemental Figure 2

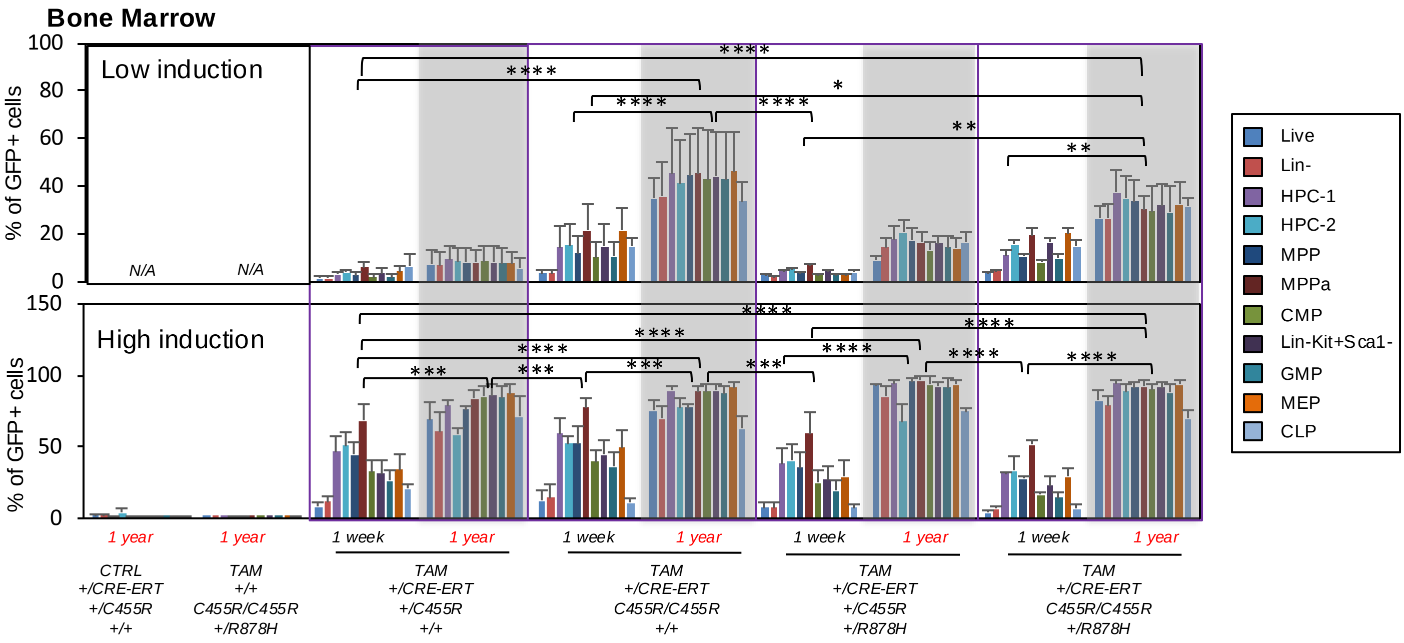

Supplemental Figure 3

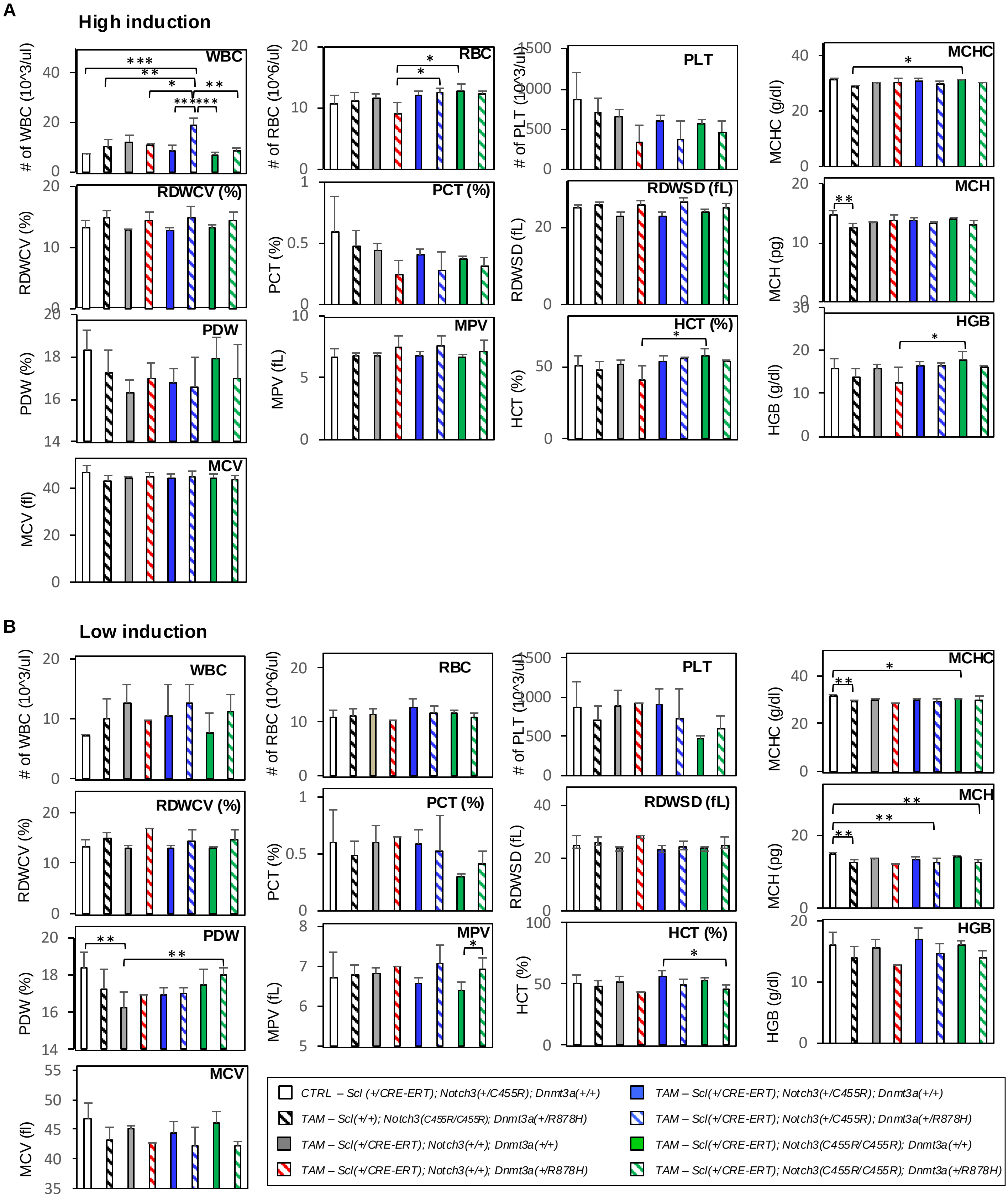

Supplemental Figure 4

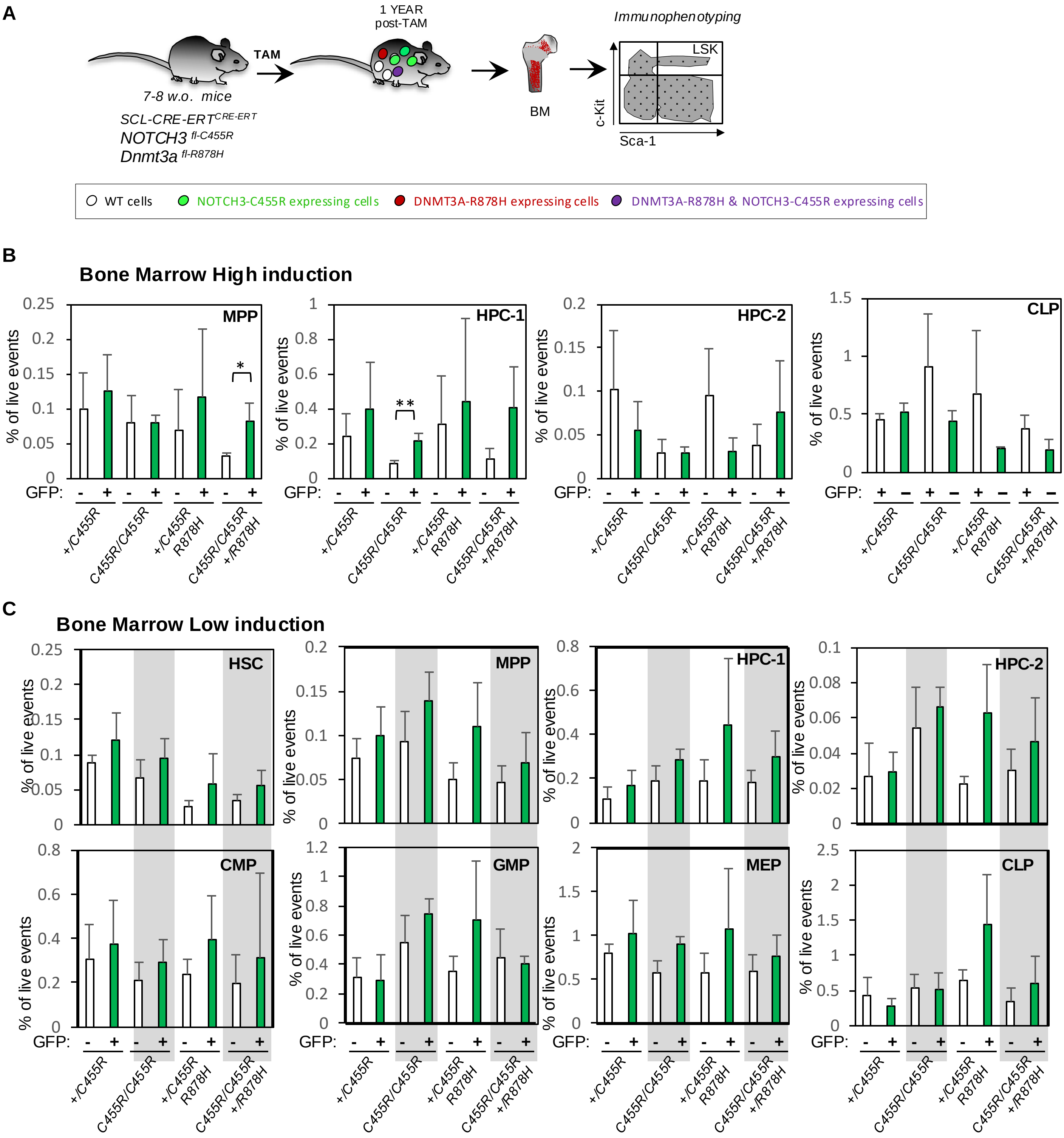

Supplemental Figure 5

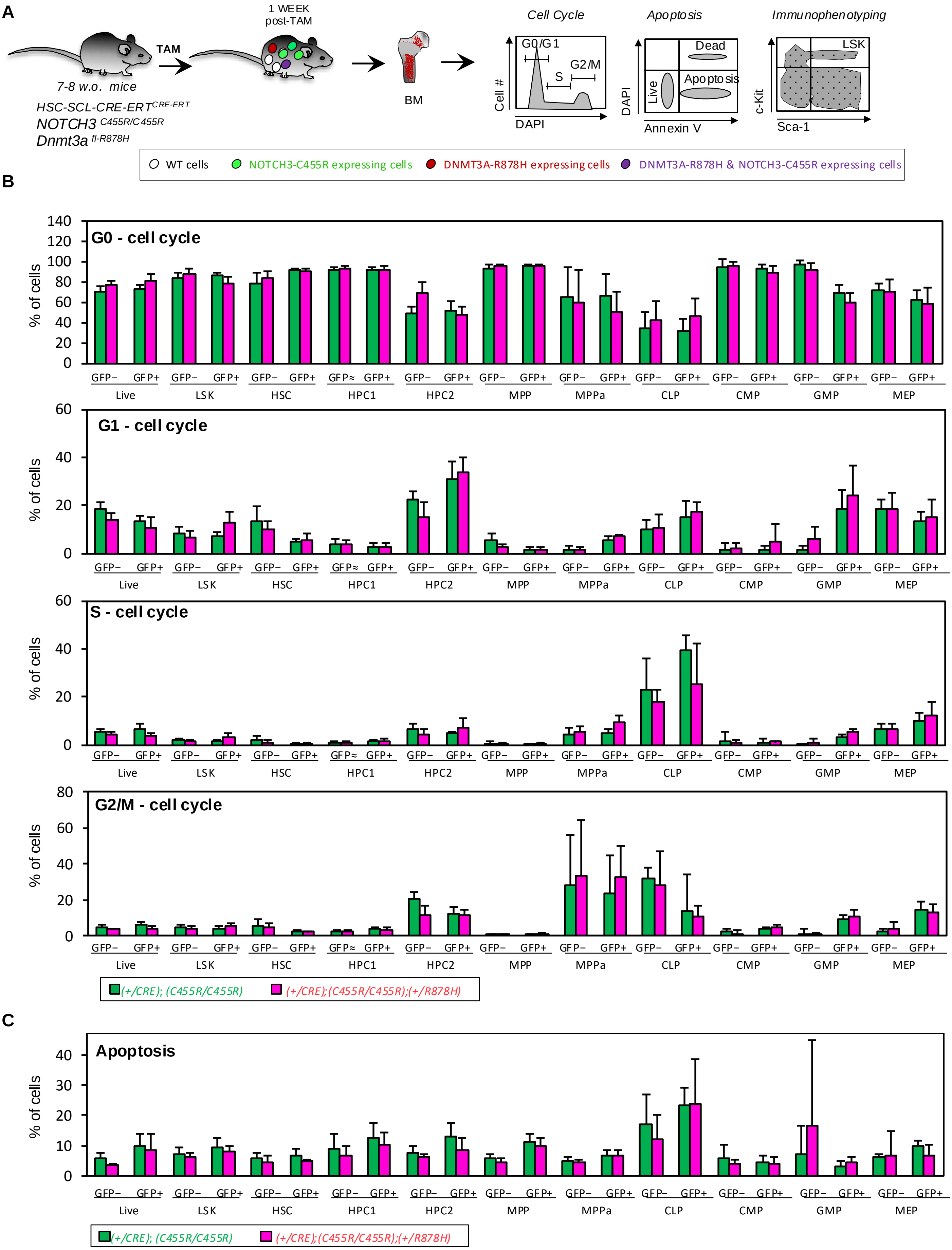

Supplemental Figure 6

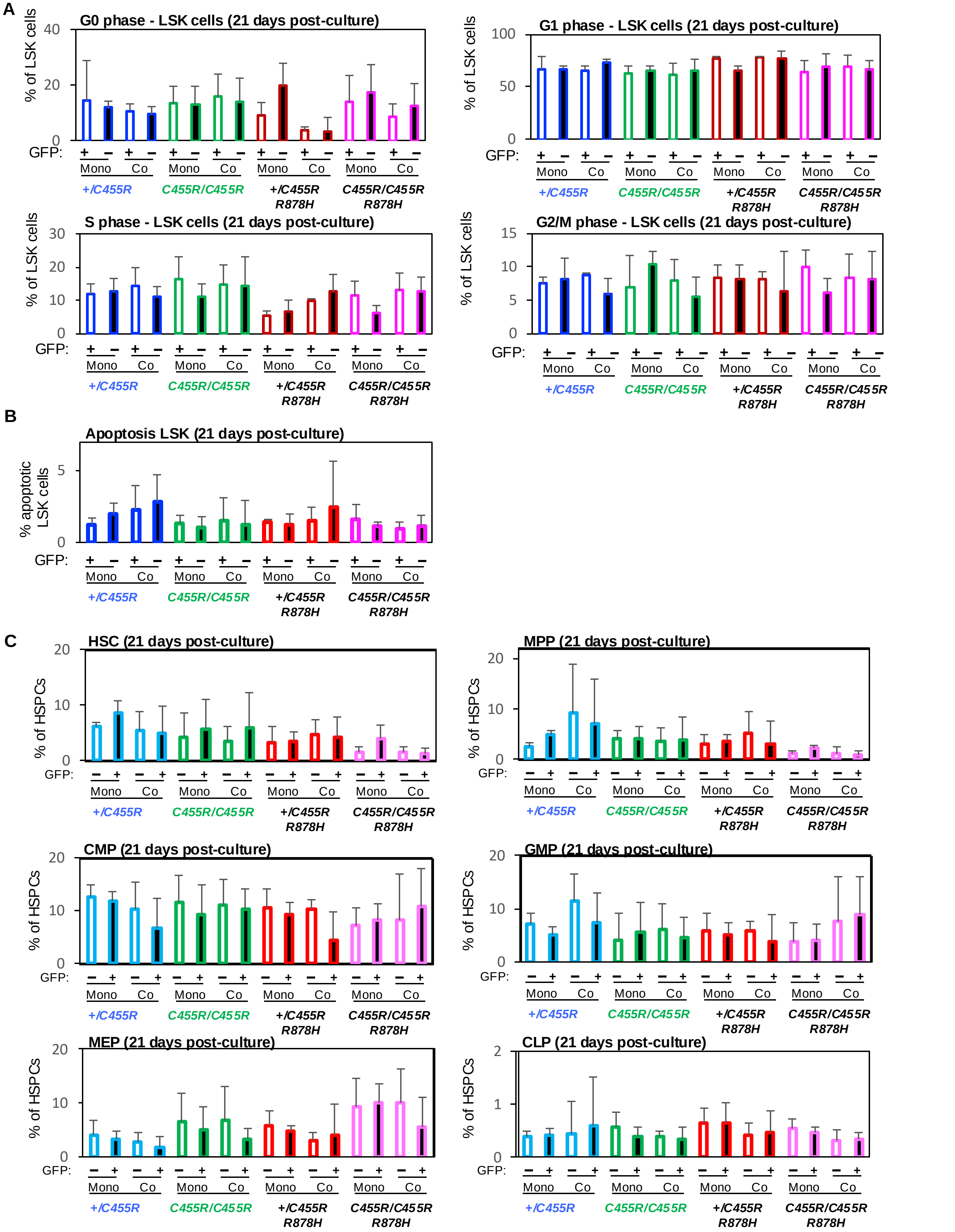

Supplemental Figure 7

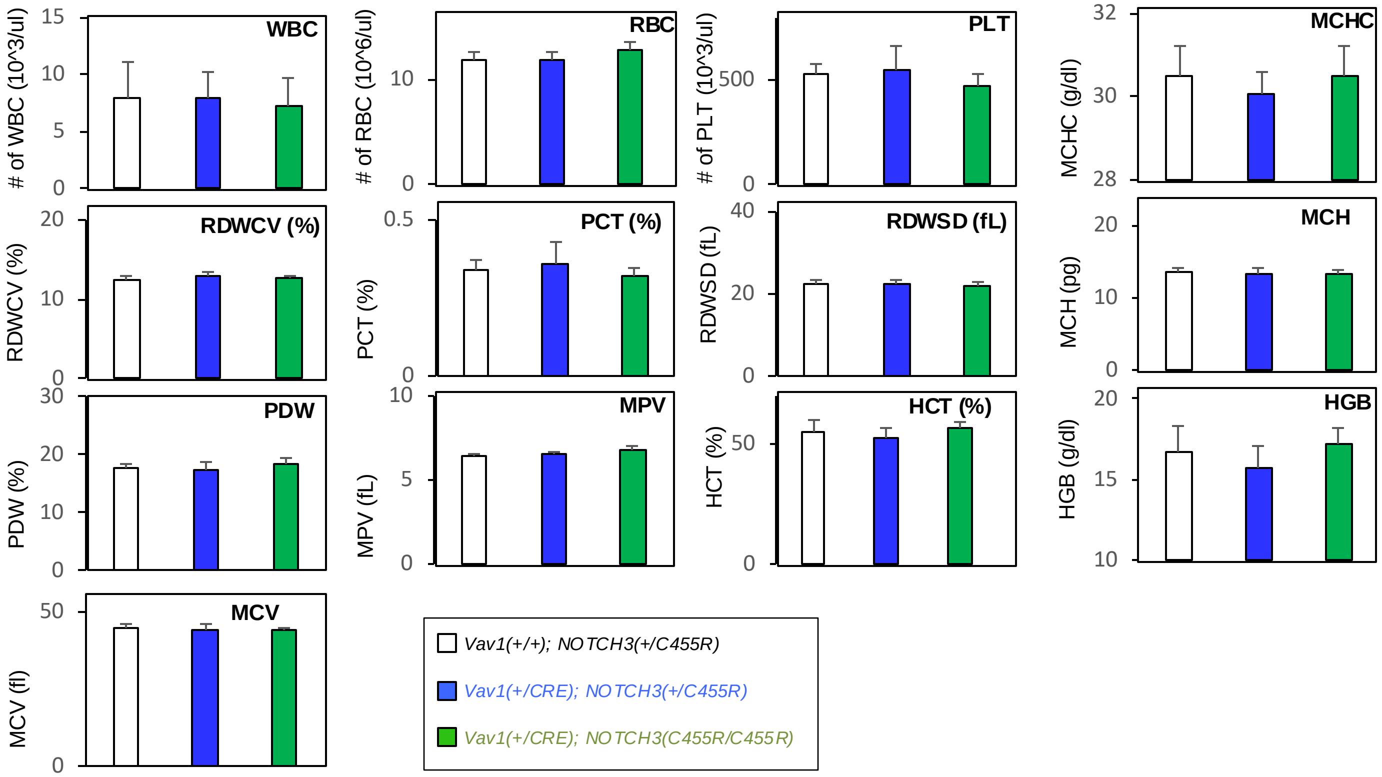

Supplemental Figure 8

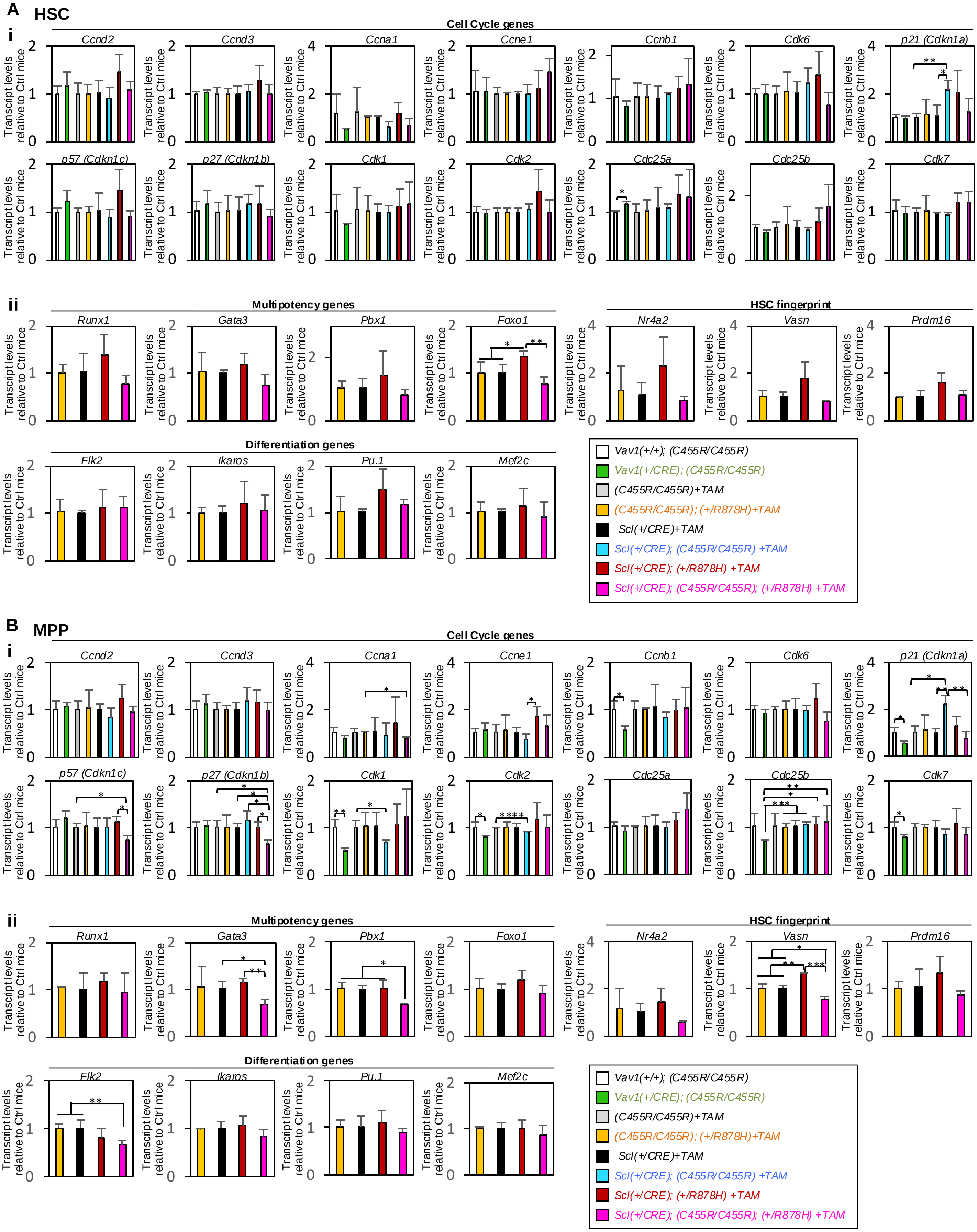

Supplemental Figure 9

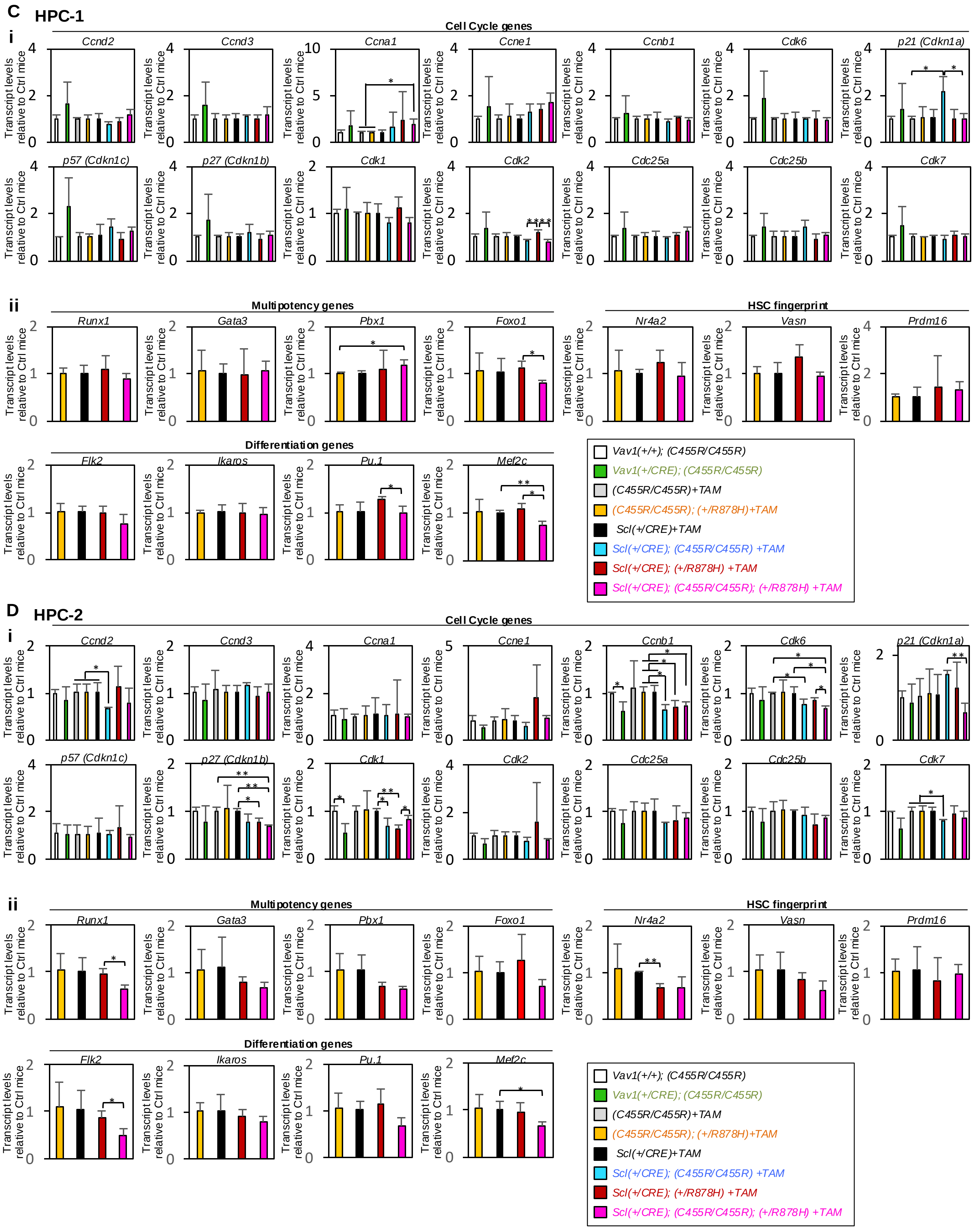

Supplemental Figure 9

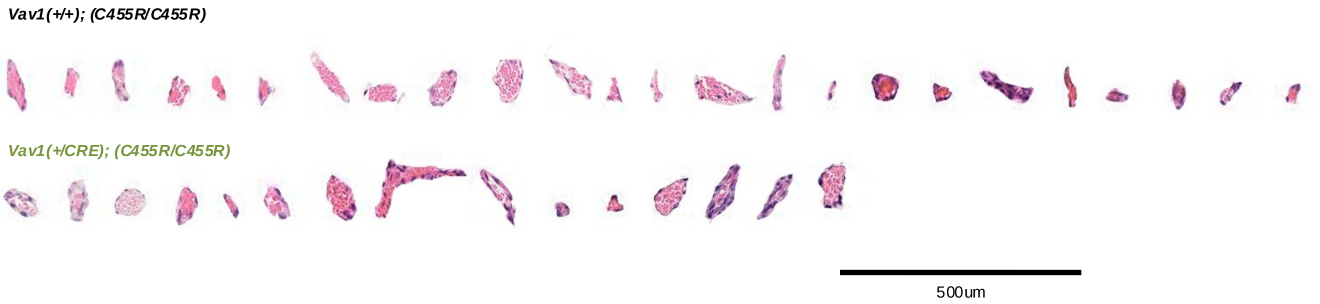

Supplemental Figure 10

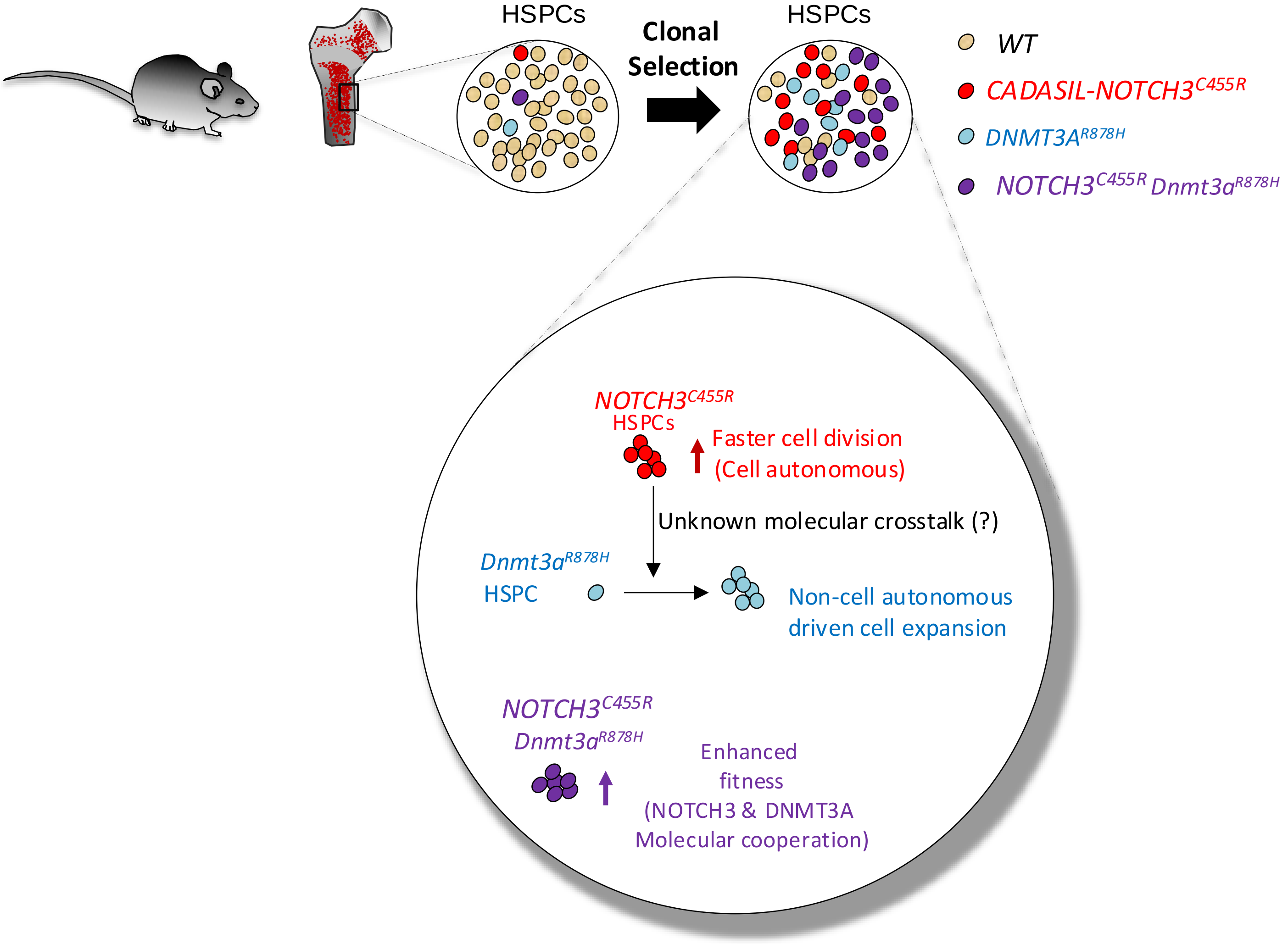

Supplemental Figure 11
